# The effect of physical fitness on psychological health: evidence from Chinese university students

**DOI:** 10.1186/s12889-024-18841-y

**Published:** 2024-05-21

**Authors:** Shuzhen Ma, Yanqi Xu, Simao Xu, Zhicheng Guo

**Affiliations:** 1 College of Public Administration, Guilin University of Technology, Guilin, 541004 China; 2 Department of Sports Studies, Faculty of Educational Studies, Universiti Putra Malaysia, Serdang, 43400 Selangor Malaysia; 3 College of Materials Science and Engineering, Key Laboratory of New Processing Technology for Nonferrous Metals and Materials, Collaborative Innovation Center for Exploration of Nonferrous Metal Deposits and Efficient Utilization of Resources, Ministry of Education, Guilin University of Technology, Guilin, 541004 China; 4 College of Sports Medicine and health, Chengdu Sports University, Chengdu, 61004 China; 5 Public physical education Department, Guangxi Arts and Crafts School, Liuzhou, 545005 China

**Keywords:** Mental health, Physical fitness, University students

## Abstract

**Background:**

Despite frequent discussions on the link between physical and mental health, the specific impact of physical fitness on mental well-being is yet to be fully established.

**Method:**

This study, carried out between January 2022 and August 2023, involved 4,484 Chinese University students from eight universities located in various regions of China. It aimed to examine the association between physical fitness on psychological well-being. Descriptive statistics, t-tests, and logistic regression were used to analyze the association between physical fitness indicators (e.g., Body Mass Index (BMI), vital capacity, and endurance running) and mental health, assessed using Symptom Checklist-90 (SCL-90). All procedures were ethically approved, and participants consented to take part in.

**Results:**

Our analysis revealed that BMI, vital capacity, and endurance running scores significantly influence mental health indicators. Specifically, a 1-point increase in BMI increases the likelihood of an abnormal psychological state by 10.9%, while a similar increase in vital capacity and endurance running decreases the risk by 2.1% and 4.1%, respectively. In contrast, reaction time, lower limb explosiveness, flexibility, and muscle strength showed no significant effects on psychological states (*p* > 0.05).

**Conclusion:**

Improvements in BMI, vital capacity, and endurance running capabilities are associated with better mental health outcomes, highlighting their potential importance in enhancing overall well-being.

**Supplementary Information:**

The online version contains supplementary material available at 10.1186/s12889-024-18841-y.

## Background

In China, while University students were traditionally perceived as being ‘blessed by fortune’ and less prone to mental distress or disorders, the rapid expansion of universities and universities during significant socio-economic transitions has brought unprecedented attention to mental health issues among this demographic in recent decades [1, 2]. Both male and female university students commonly experience psychological challenges stemming from environmental changes, academic pressures, emotional setbacks, and health issues [3]. Mental disorders during this period can lead to significant adverse outcomes, including university dropout, academic underachievement, strained relationships, and diminished emotional well-being, ultimately compromising physical health and future career prospects [4]. The Symptom Checklist-90 (SCL-90) is frequently employed in China for assessing the mental health status of university students [5]. According to Ren (2009), the SCL-90 was utilized in 63.8% of the published articles addressing mental health among university students [6].

Wang introduced the SCL-90 to China in 1984 [7]. Since its translation by Wang from English to Chinese, the scale has gained widespread usage in China [8]. It comprises 90 self-report items, with each question utilizing a 5-point Likert scale, ranging from 0 (not at all) to 4 (extremely). The SCL-90 effectively identifies individuals with existing psychiatric symptoms, screens for potential symptoms, determines their type and severity, and highlights the urgency for personalized intervention based on higher total scores [9].

Given that the World Health Organization (WHO) regards mental and physical dimensions as fundamental components of overall health and well-being [10], a robust correlation has been identified between mental and physical health [11–14]. Dr. Kishore, in an article published in the Bulletin of the WTO, notably asserted that “true physical health cannot exist without mental health” [15]. Ohrnberger (2017) discovered robust cross-effects between physical and mental health, even when adjusting for confounding variables [16]. In prior research, several cross-sectional studies have identified mental health as a significant correlate of physical health [17–19], however, there is a lack of studies investigating the dynamic association between the two [16].

Physical health, encompassing cardiorespiratory endurance, muscular strength endurance, flexibility, and body composition, serves as a critical indicator of health [20, 21]. The Ministry of Education of China released the National Physical Health Standards for Students (revised in 2014, NPHSS) to assess the physical health status of young individuals, including university students, thus reflecting their overall physical fitness level. These standards are evaluated annually, with fitness measures assessed according to the 2014 revised Chinese National Student Physical Fitness Standard (CNSPFS), covering various aspects such as aerobic capacity, upper body strength, flexibility, body mass index (BMI), abdominal strength, and trunk strength [21]. The national standards aid educators in establishing the desired objectives for students to accomplish by the conclusion of their academic endeavors [22]. Since its initial introduction, the NPHSS has been instrumental in shaping physical education policies in China. Studies have demonstrated that structured physical fitness programs, aligned with these standards, not only enhance physical health but also contribute to academic performance and psychological resilience among students. Furthermore, longitudinal data suggest that continuous engagement with NPHSS-guided activities significantly improves health outcomes over time [23].

Our study employs the 2014 revised NPHSS to assess the physical fitness level of Chinese university students, alongside the use of the SCL-90 as a screening tool for evaluating their mental health status. We analyze the discrepancy in average scores of the sports quality index between students with regular and abnormal mental health statuses and examine the impact of sports quality index scores on students’ psychological well-being using a binary logistic regression model. To provide some suggestions on how to improve the mental health status of students with abnormal mental health status in university through some physical exercises.

## Method

### Participants

Between January 2022 and August 2023, a cross-sectional study was conducted in three regions of China—North and Northeast, Northwest, and Southwest—focusing on the psychological and physical health of university students. This study was approved by the Ethics Committee of Guangxi Normal University and involved eight universities. Within each of the four academic levels at every university, 150 students were selected, totaling 4800 participants. Students were recruited on a voluntary basis from various departments within each university. A random sampling technique was applied across the different majors to ensure a representative sample, reflecting the diversity of academic disciplines. Recruitment was facilitated through university instructors in physical education and mental health, and participants were offered academic credit as an incentive, a practice approved by the ethics committee for its educational value. Recruitment and data collection were carried out from January 2022 to August 2023. Recruitment was initiated in January 2022 and completed by April 2022. Each university conducted its recruitment independently, allowing for data collection to proceed until August 2023. The participants were primarily first through fourth-year undergraduate students, typically aged 18 to 22. After excluding participants with incomplete physical fitness tests or questionnaires, data from the remaining participants were analyzed. Specifically, if a participant left any item blank or provided evidently non-serious responses (such as marking the same answer across multiple items without considering the content), we considered the data incomplete or the responses erroneous, thus excluding them from our analysis. To address the ethical consideration of incentivizing participation with academic credit, it is important to note that this practice was carefully reviewed and approved by the ethics committee. The incentive was deemed appropriate given its direct relevance to the educational outcomes of the students involved in the study. Moreover, the use of academic credit as an incentive aligns with the educational goals of the participants, enhancing their engagement in activities that contribute to their academic and personal development.

### Assessment

The research involved the evaluation of participants through a combination of physical fitness assessments and questionnaire surveys. The physical fitness evaluations adhered to the criteria outlined in the NPHSS provided by the Ministry of Education of China. These standards encompass various parameters such as BMI, lung capacity, 50-meter sprint, sit-and-reach flexibility, standing long jump, pull-ups (for men) or 1-minute sit-ups (for women), and either a 1000-meter run (for men) or an 800-meter run (for women). The physical fitness assessment greatly benefits students by assisting them in achieving higher academic credits and preparing them for the workforce with improved physical conditions.

The Symptom SCL-90 is a widely recognized tool in psychiatric assessment [24]. Its reliability has been confirmed by previous studies [25]. The scale comprises nine subscale dimensions: Somatization, Obsessive-Compulsive, Interpersonal-Sensitivity, Depression, Anxiety, Hostility, Phobic-Anxiety, Paranoid Ideation, and Psychoticism [9]. In this study, teachers from eight universities utilized mobile phones to distribute the questionnaire to their students, who were instructed to complete it accurately. The questionnaire exhibited a 100% response rate and a 94% effectiveness rate, demonstrating its utility in capturing relevant data.

### Variable table

This study integrates a thorough assessment of physical fitness encompassing seven primary dimensions. Due to inherent differences in physical characteristics between genders, variations in indicator selection are observed. The overall physical quality test comprises seven aspects: BMI, vital capacity, 50m run, standing long jump, sitting forward bend, 1000m run (male)/800m run (female), and pull-up (male)/one-minute sit-up (female). The implications of these seven indicators are delineated in Table 1 below. The ratings of ‘Excellent’, ‘Good’, ‘Pass’, and ‘Failure’ in this study are determined based on the specific scoring criteria set by the NPHSS, which adjust scoring thresholds for different physical indicators according to gender.

**Table 1:** Variable table [26]

Sport Quality Indicator	Indicator Connotation
BMI	BMI
Vital capacity	Vital capacity
50 m	Reaction rate
Standing long jump	Reflecting explosive power of lower limbs
Sitting forward	Reflecting flexibility
1000 m (male)/ 800 m (female)	Reflecting endurance running
Pull up (male)/ One-minute sit-up (female)	Reflecting muscle strength

The SCL-90 scale comprises 90 items divided into 10 distinct factors. Each factor and its corresponding items are as follows:

*Somatization* (items 1, 4, 12, 27, 40, 42, 48, 49, 52, 53, 56, and 58, totaling 12 items): Reflects distress arising from perceptions of bodily dysfunction.

*Obsessive-compulsive* (items 3, 9, 10, 28, 38, 45, 46, 51, 55, and 65, totaling 10 items): Indicates an inclination towards repetitive thoughts and compulsive behaviors.

*Interpersonal Sensitivity* (items 6, 21, 34, 36, 37, 41, 61, 69, and 73, totaling 9 items): Measures feelings of inadequacy and inferiority, particularly in comparison to others.

*Depression* (items 5, 14, 15, 20, 22, 26, 29, 30, 31, 32, 54, 71, and 79, totaling 13 items): Assesses symptoms associated with mood disturbance, including melancholy, hopelessness, and the lack of interest in life.

*Anxiety* (items 2, 17, 23, 33, 39, 57, 72, 78, 80, and 86, totaling 10 items): Evaluates general signs of anxiety such as nervousness, tension, and tremulousness.

*Hostility* (items 11, 24, 63, 67, 74, and 81, totaling 6 items): Concerns feelings of anger and irritability.

*Phobic Anxiety* (items 13, 25, 47, 50, 70, 75, and 82, totaling 7 items): Represents persistent and irrational fears about specific objects, people, or situations.

*Paranoid Ideation* (items 8, 18, 43, 68, 76, and 83, totaling 6 items): Involves thoughts or beliefs of being harmed by others.

*Psychoticism* (items 7, 16, 35, 62, 77, 84, 85, 87, 88, and 90, totaling 10 items): Encompasses a range of symptoms suggestive of psychosis, including isolation and withdrawal.

*Other* (items 19, 44, 59, 60, 64, 66, and 89, totaling 7 items): Items that do not neatly fall into the above categories.

Each item employs a 5-level scoring system with the following instructions: None (1 point), Very mild (2 points), Moderate (3 points), Severe (4 points), and very severe (5 points). Factor scores are computed by summing the scores of each item within the factor and dividing by the number of items within that factor. A score of 1–2 indicates a normal result, while a score greater than 2 indicates an abnormal result.

### Equity, diversity and inclusion

This research targeted university students across China, with recruitment strategies meticulously designed to accommodate the accessibility requirements, geographical diversity, educational attainment, and socioeconomic statuses of participants. To achieve a balanced and diverse sample, recruitment efforts spanned multiple provinces, deliberately including individuals of varying genders and ethnic backgrounds. The composition of the author team reflects a commitment to diversity, evidenced by a gender balance and a wide array of research disciplines represented.

### Data analysis

This study conducted descriptive statistics analysis on the total score of Sport Quality test and SCL-90 scale test results of the total sample, respectively; And according to gender differences, descriptive statistics analysis was also conducted for the total score of Sport Quality test and SCL-90 scale test results. This study uses statistical software SPSS for Independent sample t-test and logistic regression analysis. Specifically, independent sample t test was used to compare differences between psychological state among seven Sport Quality indicators. Logistic regression analysis was used to assess the impacts of scores of sport quality indicators on students’ psychological state. In the logistic regression analysis, this study used the 10 factors (somatization, obsessive-compulsive, interpersonal sensitivity, depression, anxiety, hostility, phobic anxiety, paranoid ideation, psychoticism and others) in the SCL-90 scale as the dependent variables and the scores of the seven aspects (BMI, vital capacity, reaction rate, lower limb explosive force, flexibility, endurance running and muscle strength) of physical testing as the independent variables for regression analysis. The dependent variable is divided into two categories: normal (record as 1) and abnormal (record as 2), this study uses the binary logistic regression model to explore the impacts of scores of sport quality indicators on students’ psychological state. Making adjustments in logistic regression involves several crucial steps, including variable selection, model diagnostics, and validation to ensure robustness and relevance. The variable selection method of the manual selection based on theoretical understanding was used in the current study. Variables with a high p-value (above a threshold, typically 0.05) are considered insignificant and can be excluded. This study uses tests and plots to check for the adequacy of the model fit.

## Results

In the physical fitness assessments, adjustments were made to account for potential confounders such as weather conditions and venue characteristics. These variables were selected due to their potential impact on the physical performance outcomes measured in the study. By controlling for these environmental and situational variables during the testing phase, we aimed to ensure that the data on sport quality indicators accurately reflect the students’ physical capabilities, minimizing any external influences that could affect the outcomes. In this study, our initial target was to include 4,800 university students. However, a total of 4,484 students participated in the physical and psychological assessments, leading to a participation rate of 9%. The primary reasons for non-participation included absenteeism on the scheduled days of testing and incomplete survey responses. We estimated the required sample size using G*Power software, based on an effect size of 0.138 derived from previous studies [27]. The analysis indicated that a minimum sample size of 1832 was necessary. Our actual sample size of 4484 significantly exceeds this threshold, confirming the statistical robustness of our findings.

### Descriptive statistics of total score of sport quality test results

In 2023, a total of 4,484 university students in China participated in this study, comprising 3,565 males and 919 females, who underwent physical and psychological testing. The results of the sports quality test are presented in Table 2. It shows that only five students achieved an ‘excellent’ total score, representing a mere 0.1% of participants. Additionally, 268 students scored ‘good,’ accounting for 6.0% of the total; 3,731 students received a ‘pass,’ making up 83.2% and representing the largest proportion; and 480 students were categorized as ‘fail,’ comprising 10.7% of the total.

**Table 2:** Descriptive statistics of total score of sport quality test

Total Score of Sport Quality Test	Overall	Boys	Girls
Excellent	5 (0.1%)	1 (0.0%)	4 (0.4%)
Good	268 (6.0%)	207 (5.8%)	61 (6.6%)
Pass	3731(83.2%)	2893 (81.2%)	838 (91.2%)
Failure	480 (10.7%)	464 (13.0%)	16 (1.7%)
Total	4484 (100%)	3565 (100%)	919 (100%)

### Descriptive statistics of SCL-90 scale test results

Table 3 displays the frequency distribution of SCL-90 scale test results, indicating that a higher number of students exhibited normal psychological outcomes compared to those with abnormal results, representing 8.7% of the total sample. Among the students, 8.5% of males and 9.5% of females showed abnormal psychological test outcomes. The predominant dimensions observed in the overall sample were obsessive-compulsive disorder (13.4%), interpersonal sensitivity (9.3%), and depression (8.3%), with somatization presenting the lowest incidence at 4.6%. For males, the primary issues were obsessive-compulsive disorder (13.0%), interpersonal sensitivity (9.3%), and depression (7.8%), with somatization at 4.5%. Among females, the main dimensions identified were obsessive-compulsive disorder (14.8%), interpersonal sensitivity (9.5%), and depression (10.3%), with somatization at 5.0%.

**Table 3:** Descriptive statistics of SCL-90 scale test results

		Overall		Boys		Girls	
		Frequency	Percent (%)	Frequency	Percent (%)	Frequency	Percent (%)
Somatization	Normal	4278	95.4	3405	95.5	873	95.0
	Abnormal	206	4.6	160	4.5	46	5.0
Obsessive-Compulsive	Normal	3884	86.6	3101	87.0	783	85.2
	Abnormal	600	13.4	464	13.0	136	14.8
Interpersonal Sensitivity	Normal	4065	90.7	3233	90.7	832	90.5
	Abnormal	419	9.3	332	9.3	87	9.5
Depression	Normal	4111	91.7	3287	92.2	824	89.7
	Abnormal	373	8.3	278	7.8	95	10.3
Anxiety	Normal	4205	93.8	3351	94.0	854	92.9
	Abnormal	279	6.2	214	6.0	65	7.1
Hostility	Normal	4220	94.1	3354	94.1	866	94.2
	Abnormal	264	5.9	211	5.9	53	5.8
Phobic Anxiety	Normal	4241	94.6	3373	94.6	868	94.5
	Abnormal	243	5.4	192	5.4	51	5.5
Paranoid Ideation	Normal	4212	93.9	3340	93.7	872	94.9
	Abnormal	272	6.1	225	6.3	47	5.1
Psychoticism	Normal	4199	93.6	3334	93.5	865	94.1
	Abnormal	285	6.4	231	6.5	54	5.9
Others	Normal	4146	92.5	3291	92.3	855	93.0
	Abnormal	338	7.5	274	7.7	64	7.0
Mean total score	Normal	4095	91.3	3263	91.5	832	90.5
	Abnormal	389	8.7	302	8.5	87	9.5
Total		4484	100.0	3565	100.0	919	100.0

### Differences of sport quality indicators between normal and abnormal state of psychological test (*N* = 4484)

Table 4 displays the differences in sport quality indicators between students with normal and abnormal psychological test states. Across the overall sample, as well as separately within the male and female groups, the mean BMI was significantly lower in those with a normal psychological state compared to those with an abnormal state (*p* < 0.01). Additionally, the mean scores for vital capacity and endurance running were significantly higher in the normal psychological state than in the abnormal state (*p* < 0.01). However, no significant differences were observed in the sport quality indicators of reaction rate, lower limb explosive force, flexibility, and muscle strength between the two groups (*p* > 0.05).

**Table 4:** Differences of Sport Quality Indicators Between Normal and Abnormal State of Psychological Test (*N* = 4484)

	Overall Psychology State (*N* = 4484)				Overall Psychology State of Boys (*N* = 3565)				Overall Psychology State of Girls (*N* = 919)			
	Normal	Abnormal	*p* value	95% CI	Normal	Abnormal	*p* value	95% CI	Normal	Abnormal	*p* value	95% CI
BMI	65.15 ± 9.35	88.59 ± 15.38	< 0.001	(-24.99, -21.87)	65.59 ± 9.48	87.22 ± 15.89	< 0.001	(-23.46, -19.80)	63.44 ± 8.62	93.33 ± 12.45	< 0.001	(-32.61, -27.18)
Vital capacity	69.51 ± 12.99	59.90 ± 17.89	< 0.001	(7.78, 11.44)	69.41 ± 13.28	59.54 ± 17.69	< 0.001	(7.82, 11.93)	69.89 ± 11.79	61.17 ± 18.64	< 0.001	(4.67, 12.77)
Reaction rate	69.25 ± 8.69	69.16 ± 8.44	0.833	(-0.80, 1.00)	71.01 ± 6.50	71.13 ± 5.80	0.754	(-0.88, 0.64)	62.36 ± 12.10	62.30 ± 11.94	0.962	(-2.61, 2.74)
Lower limb explosive force	62.99 ± 17.85	63.68 ± 18.37	0.465	(-2.55, 1.17)	61.65 ± 18.35	62.23 ± 19.20	0.603	(-2.75, 1.60)	68.22 ± 14.61	68.71 ± 14.12	0.763	(-3.72, 2.73)
Flexibility	72.96 ± 16.30	72.27 ± 16.52	0.425	(-1.01, 2.39)	71.50 ± 16.43	71.05 ± 16.42	0.647	(-1.49, 2.39)	78.68 ± 14.44	76.51 ± 16.23	0.187	(-1.06, 5.41)
Endurance Running	73.44 ± 9.66	56.55 ± 20.61	< 0.001	(14.82, 18.97)	73.63 ± 9.67	57.63 ± 20.36	< 0.001	(13.67, 18.33)	72.72 ± 9.59	52.82 ± 21.13	< 0.001	(15.36, 24.46)
Muscle strength	40.42 ± 32.04	40.83 ± 31.43	0.809	(-374, 2.92)	34.11 ± 32.56	33.10 ± 31.36	0.605	(-2.82, 4.84)	65.17 ± 11.20	67.67 ± 8.82	0.045	(-4.93, -0.06)

### Logistic regression analysis

In order to explore the impact of physical fitness on psychological health of university students from a quantitative perspective. This study used the 10 factors (somatization, obsessive-compulsive, interpersonal sensitivity, depression, anxiety, hostility, phobic anxiety, paranoid ideation, psychoticism and others) in the SCL-90 scale as the dependent variables and the scores of the seven aspects (BMI, vital capacity, reaction rate, lower limb explosive force, flexibility, endurance running and muscle strength) of physical testing as the independent variables for regression analysis. In this study, because the dependent variables (overall psychological state, somatization, obsessive-compulsive, interpersonal sensitivity, depression, anxiety, hostility, phobic anxiety, paranoid ideation, psychoticism and others) are divided into two categories: normal (record as 1) and abnormal (record as 2), this study uses the binary logistic regression models to explore the impacts of scores of sport quality indicators (BMI, vital capacity, reaction rate, lower limb explosive force, flexibility, endurance running and muscle strength) on students’ psychological state. The results are shown in Table 5, 6 and 7.

Firstly, in binary logistic regression, the overall psychological state is taken as the dependent variable. It can be seen from Table 5 that the regression coefficients of the independent variables, BMI (*p* < 0.01), vital capacity (*p* < 0.01) and endurance running (*p* < 0.01) were significant, while the regression coefficients of the other four independent variables reaction rate (*p* > 0.05), lower limb explosive force (*p* > 0.05), flexibility (*p* > 0.05) and muscle strength (*p* > 0.05) were not significant. From this, it can be concluded that there are three factors that affect students’ psychological state in the overall sample, namely, BMI, vital capacity, and endurance running. From Table 5, it can be further seen that for every 1 point increase in BMI score, the risk of students’ psychological state being abnormal increases by 10.9%; when the vital capacity score increases by 1 point, the risk of a student’s psychological state being abnormal decreases by 2.1%; when the endurance running score increases by 1 point, the risk of a student’s psychological state being abnormal decreases by 4.1%.

Next, in binary logistic regression, the factor of somatization is taken as the dependent variable. From Table 5, it is observed that the regression coefficients of the independent variables, BMI (*p* < 0.01), vital capacity (*p* < 0.01) and endurance running (*p* < 0.01) were significant, while the regression coefficients of the other four independent variables reaction rate (*p* > 0.05), lower limb explosive force (*p* > 0.05), flexibility (*p* > 0.05) and muscle strength (*p* > 0.05) were not significant. From this, it can be concluded that there are three factors that affect students’ somatization in the overall sample, namely, BMI, vital capacity and endurance running. From Table 5, it can be further seen that for every 1 point increase in BMI score, the risk of students’ somatization being abnormal increases by 8.8%; when the vital capacity score increases by 1 point, the risk of a student’s somatization being abnormal decreases by 1.8%; when the endurance running score increases by 1 point, the risk of a student’s somatization being abnormal decreases by 2.7%.

In the binary logistic regression, the factor of obsessive-compulsive is taken as the dependent variable. It can be seen from Table 5 that the regression coefficients of the independent variables, BMI (*p* < 0.01), vital capacity (*p* < 0.01) and endurance running (*p* < 0.01) were significant, while the regression coefficients of the other four independent variables reaction rate (*p* > 0.05), lower limb explosive force (*p* > 0.05), flexibility (*p* > 0.05) and muscle strength (*p* > 0.05) were not significant. From this, it can be concluded that there are three factors that affect students’ obsessive-compulsive in the overall sample, namely, BMI, vital capacity, and endurance running. From Table 5, it can be further seen that for every 1-point increase in BMI score, the risk of students’ obsessive-compulsive being abnormal increases by 7.8%; when the vital capacity score increases by 1 point, the risk of a student’s obsessive-compulsive being abnormal decreases by 2.0%; when the endurance running score increases by 1 point, the risk of a student’s obsessive-compulsive being abnormal decreases by 3.0%.

In the binary logistic regression, the factor of interpersonal sensitivity is taken as the dependent variable. It can be seen from Table 5 that the regression coefficients of the independent variables, BMI (*p* < 0.01), vital capacity (*p* < 0.01) and endurance running (*p* < 0.01) were significant, while the regression coefficients of the other four independent variables reaction rate (*p* > 0.05), lower limb explosive force (*p* > 0.05), flexibility (*p* > 0.05) and muscle strength (*p* > 0.05) were not significant. From this, it can be concluded that there are three factors that affect students’ interpersonal sensitivity in the overall sample, namely, BMI, vital capacity, and endurance running. From Table 5, it can be further seen that for every 1 point increase in BMI score, the risk of students’ interpersonal sensitivity being abnormal increases by 9.2%; when the vital capacity score increases by 1 point, the risk of a student’s interpersonal sensitivity being abnormal decreases by 1.6%; when the endurance running score increases by 1 point, the risk of a student’s interpersonal sensitivity being abnormal decreases by 3.1%.

**Table 5:** Results of Sport Quality Indicators Logistic Regression Analysis Between Normal and Abnormal Score of Psychological Test (*N* = 4484)

	Dependent variable: overall psychological state					Dependent variable: Somatization					Dependent variable: Obsessive-Compulsive					Dependent variable: Interpersonal Sensitivity				
	B	*p*	OR	95% CI		B	*p*	OR	95% CI		B	*p*	OR	95% CI		B	*p*	OR	95% CI	
				Lower	Upper				Lower	Upper				Lower	Upper				Lower	Upper
BMI	0.109	< 0.001	1.116	1.104	1.128	0.088	< 0.001	1.092	1.079	1.106	0.078	< 0.001	1.081	1.072	1.089	0.092	< 0.001	1.096	1.086	1.106
Vital capacity	-0.021	< 0.001	0.979	0.970	0.988	-0.018	< 0.001	0.982	0.972	0.992	-0.020	< 0.001	0.980	0.973	0.988	-0.016	< 0.001	0.984	0.975	0.992
Reaction rate	0.004	0.639	1.004	0.986	1.022	0.003	0.757	1.003	0.982	1.026	0.003	0.619	1.003	0.990	1.017	0.008	0.336	1.008	0.992	1.025
Lower limb explosive force	0.005	0.247	1.005	0.996	1.014	0.011	0.077	1.011	0.999	1.023	0.005	0.145	1.005	0.998	1.012	0.008	0.073	1.008	0.999	1.016
Flexibility	-0.001	0.738	0.999	0.990	1.007	0.004	0.457	1.004	0.993	1.015	0.000	0.918	1.000	0.993	1.006	-0.001	0.806	0.999	0.992	1.007
Endurance Running	-0.041	< 0.001	0.960	0.949	0.970	-0.027	< 0.001	0.973	0.964	0.983	-0.030	< 0.001	0.970	0.962	0.979	-0.031	< 0.001	0.969	0.960	0.978
Muscle strength	0.001	0.536	1.001	0.997	1.006	0.003	0.334	1.003	0.997	1.008	0.000	0.985	1.000	0.997	1.003	0.000	0.880	1.000	0.996	1.004
(Constant)	-6.958	< 0.001	0.001			-8.103	< 0.001	0.000			-4.536	< 0.001	0.011			-6.792	< 0.001	0.001		

B represents the non-standardized coefficient, P represents the significance, OR represents the odds ratio and 95% CI represents the 95% confidence interval

In the binary logistic regression, the factor of depression is taken as the dependent variable. It can be seen from Table 6 that the regression coefficients of the independent variables, vital capacity (*p* < 0.01) and endurance running (*p* < 0.01) were significant, while the regression coefficients of the other five independent variables BMI (*p* > 0.05), reaction rate (*p* > 0.05), lower limb explosive force (*p* > 0.05), flexibility (*p* > 0.05) and muscle strength (*p* > 0.05) were not significant. From this, it can be concluded that there are two factors that affect students’ depression in the overall sample, namely, vital capacity and endurance running. From Table 6, it can be further seen that when the vital capacity score increases by 1 point, the risk of a student’s depression being abnormal decreases by 5.6%; when the endurance running score increases by 1 point, the risk of a student’s depression being abnormal decreases by 16.5%.

In the binary logistic regression, the factor of anxiety is taken as the dependent variable. It can be seen from Table 6 that the regression coefficients of the independent variables, BMI (*p* < 0.01), vital capacity (*p* < 0.01), lower limb explosive force (*p* < 0.05) and endurance running (*p* < 0.01) were significant, while the regression coefficients of the other three independent variables reaction rate (*p* > 0.05), flexibility (*p* > 0.05) and muscle strength (*p* > 0.05) were not significant. From this, it can be concluded that there are four factors that affect students’ anxiety in the overall sample, namely, BMI, vital capacity, lower limb explosive force and endurance running. From Table 6, it can be further seen that for every 1 point increase in BMI score, the risk of students’ anxiety being abnormal increases by 8.3%; when the vital capacity score increases by 1 point, the risk of a student’s anxiety being abnormal decreases by 1.9%; when the lower limb explosive force score increases by 1 point, the risk of a student’s anxiety being abnormal increases by 2.0%;when the endurance running score increases by 1 point, the risk of a student’s anxiety being abnormal decreases by 1.7%.

In the binary logistic regression, the factor of hostility is taken as the dependent variable. It can be seen from Table 6 that the regression coefficients of the independent variables, BMI (*p* < 0.01), vital capacity (*p* < 0.01) and endurance running (*p* < 0.01) were significant, while the regression coefficients of the other four independent variables reaction rate (*p* > 0.05), lower limb explosive force (*p* > 0.05), flexibility (*p* > 0.05) and muscle strength (*p* > 0.05) were not significant. From this, it can be concluded that there are three factors that affect students’ hostility in the overall sample, namely, BMI, vital capacity and endurance running. From Table 6, it can be further seen that for every 1 point increase in BMI score, the risk of students’ hostility being abnormal increases by 8.8%; when the vital capacity score increases by 1 point, the risk of a student’s hostility being abnormal decreases by 1.5%; when the endurance running score increases by 1 point, the risk of a student’s hostility being abnormal decreases by 2.3%.

In the binary logistic regression, the factor of phobic anxiety is taken as the dependent variable. It can be seen from Table 6 that the regression coefficients of the independent variables, BMI (*p* < 0.01) and endurance running (*p* < 0.01) were significant, while the regression coefficients of the other five independent variables vital capacity (*p* > 0.05), reaction rate (*p* > 0.05), lower limb explosive force (*p* > 0.05), flexibility (*p* > 0.05) and muscle strength (*p* > 0.05) were not significant. From this, it can be concluded that there are two factors that affect students’ phobic anxiety in the overall sample, namely, BMI and endurance running. From Table 6, it can be further seen that for every 1 point increase in BMI score, the risk of students’ phobic anxiety being abnormal increases by 9.5%; when the endurance running score increases by 1 point, the risk of a student’s phobic anxiety being abnormal decreases by 1.7%.

**Table 6:** Results of sport quality indicators logistic regression analysis between normal and abnormal score of psychological Test (*N* = 4484)

	Dependent variable: Depression					Dependent variable: Anxiety					Dependent variable: Hostility					Dependent variable: Phobic Anxiety				
	B	*p*	OR	95% CI		B	*p*	OR	95% CI		B	*p*	OR	95% CI		B	*p*	OR	95% CI	
				Lower	Upper				Lower	Upper				Lower	Upper				Lower	Upper
BMI	1.040	0.970	2.828	0.000	7.E + 23	0.083	< 0.001	1.086	1.066	1.107	0.088	< 0.001	1.092	1.081	1.105	0.095	< 0.001	1.100	1.087	1.113
Vital capacity	-0.056	< 0.001	0.945	0.926	0.965	-0.019	0.004	0.981	0.968	0.994	-0.015	0.001	0.985	0.976	0.994	-0.009	0.073	0.991	0.982	1.001
Reaction rate	0.005	0.825	1.005	0.965	1.046	-0.013	0.367	0.987	0.960	1.015	-0.005	0.600	0.995	0.978	1.013	-0.002	0.876	0.999	0.980	1.018
Lower limb explosive force	-0.003	0.737	0.997	0.980	1.015	0.020	0.022	1.021	1.003	1.039	0.001	0.903	1.001	0.992	1.010	0.009	0.096	1.009	0.998	1.019
Flexibility	-0.003	0.692	0.997	0.980	1.013	-0.012	0.080	0.988	0.976	1.001	-0.001	0.869	0.999	0.990	1.008	-0.002	0.721	0.998	0.989	1.008
Endurance Running	-0.165	< 0.001	0.848	0.814	0.883	-0.017	0.008	0.983	0.970	0.996	-0.023	< 0.001	0.978	0.969	0.986	-0.017	< 0.001	0.983	0.974	0.992
Muscle strength	0.000	0.960	1.000	0.990	1.010	-0.005	0.229	0.995	0.987	1.003	-0.001	0.706	0.999	0.994	1.004	0.000	0.966	1.000	0.995	1.005
(Constant)	-71.066	0.974	0.000			-7.389	< 0.001	0.001			-6.512	< 0.001	0.001			-8.665	< 0.001	0.000		

B represents the non-standardized coefficient, P represents the significance, OR represents the odds ratio and 95% CI represents the 95% confidence interval

In the binary logistic regression, the factor of paranoid ideation is taken as the dependent variable. It can be seen from Table 7 that the regression coefficients of the independent variables, BMI (*p* < 0.01), vital capacity (*p* < 0.01) and endurance running (*p* < 0.01) were significant, while the regression coefficients of the other four independent variables reaction rate (*p* > 0.05), lower limb explosive force (*p* > 0.05), flexibility (*p* > 0.05) and muscle strength (*p* > 0.05) were not significant. From this, it can be concluded that there are three factors that affect students’ paranoid ideation in the overall sample, namely, BMI, vital capacity and endurance running. From Table 7, it can be further seen that for every 1 point increase in BMI score, the risk of students’ paranoid ideation being abnormal increases by 10.4%; when the vital capacity score increases by 1 point, the risk of a student’s paranoid ideation being abnormal decreases by 1.4%; when the endurance running score increases by 1 point, the risk of a student’s paranoid ideation being abnormal decreases by 1.9%.

In the binary logistic regression, the factor of psychoticism is taken as the dependent variable. It can be seen from Table 7 that the regression coefficients of the independent variables, BMI (*p* < 0.01), vital capacity (*p* < 0.01) and endurance running (*p* < 0.01) were significant, while the regression coefficients of the other four independent variables reaction rate (*p* > 0.05), lower limb explosive force (*p* > 0.05), flexibility (*p* > 0.05) and muscle strength (*p* > 0.05) were not significant. From this, it can be concluded that there are three factors that affect students’ psychoticism in the overall sample, namely, BMI, vital capacity and endurance running. From Table 7, it can be further seen that for every 1 point increase in BMI score, the risk of students’ psychoticism being abnormal increases by 11.0%; when the vital capacity score increases by 1 point, the risk of a student’s psychoticism being abnormal decreases by 1.9%; when the endurance running score increases by 1 point, the risk of a student’s psychoticism being abnormal decreases by 2.0%.

In the binary logistic regression, the factor of Others is taken as the dependent variable. It can be seen from Table 7 that the regression coefficients of the independent variables, BMI (*p* < 0.01), vital capacity (*p* < 0.01) and endurance running (*p* < 0.01) were significant, while the regression coefficients of the other four independent variables reaction rate (*p* > 0.05), lower limb explosive force (*p* > 0.05), flexibility (*p* > 0.05) and muscle strength (*p* > 0.05) were not significant. From this, it can be concluded that there are three factors that affect students’ Others in the overall sample, namely, BMI, vital capacity and endurance running. From Table 7, it can be further seen that for every 1 point increase in BMI score, the risk of students’ Others being abnormal increases by 8.3%; when the vital capacity score increases by 1 point, the risk of a student’s Others being abnormal decreases by 1.6%; when the endurance running score increases by 1 point, the risk of a student’s Others being abnormal decreases by 2.7%.

**Table 7:** Results of sport quality indicators logistic regression analysis between normal and abnormal score of psychological Test (*N* = 4484)

	Dependent variable: Paranoid Ideation					Dependent variable: Psychoticism					Dependent variable: Others				
	B	*p*	OR	95% CI		B	*p*	OR	95% CI		B	*p*	OR	95% CI	
				Lower	Upper				Lower	Upper				Lower	Upper
BMI	0.104	< 0.001	1.110	1.097	1.123	0.110	< 0.001	1.116	1.103	1.129	0.083	< 0.001	1.086	1.076	1.097
Vital capacity	-0.014	0.003	0.986	0.977	0.995	-0.019	< 0.001	0.982	0.972	0.991	-0.016	< 0.001	0.984	0.976	0.993
Reaction rate	0.007	0.473	1.007	0.988	1.027	-0.001	0.905	0.999	0.980	1.018	0.014	0.146	1.014	0.995	1.032
Lower limb explosive force	-0.003	0.570	0.997	0.988	1.007	0.007	0.138	1.007	0.998	1.017	0.000	0.934	1.000	0.992	1.009
Flexibility	-0.002	0.736	0.998	0.989	1.008	-0.005	0.243	0.995	0.986	1.004	0.005	0.223	1.005	0.997	1.014
Endurance Running	-0.019	< 0.001	0.981	0.972	0.990	-0.020	< 0.001	0.980	0.971	0.990	-0.027	< 0.001	0.973	0.964	0.982
Muscle strength	-0.001	0.637	0.999	0.994	1.004	-0.003	0.198	0.997	0.992	1.002	0.001	0.805	1.001	0.996	1.005
(Constant)	-8.618	< 0.001	0.000			-8.405	< 0.001	0.000			-7.112	< 0.001	0.001		

B represents the non-standardized coefficient, P represents the significance, OR represents the odds ratio and 95% CI represents the 95% confidence interval

## Discussion

This study reveals a potential correlation between physical fitness and mental health, highlighting the beneficial effects of lowering BMI, enhancing lung capacity, and engaging in endurance running on various aspects of mental well-being, such as somatization, obsessive tendencies, interpersonal sensitivity, depression, anxiety, hostility, phobic anxiety, paranoid ideation, and psychosis. These findings offer valuable insights into strategies for enhancing overall health and well-being.

In our study, we found that for every one-point increase in BMI, students face an 8.8% higher risk of abnormal somatization, a 7.8% higher risk of obsessive-compulsive disorder, a 9.2% higher risk of abnormal interpersonal sensitivity, an 8.3% higher risk of abnormal anxiety, an 8.8% higher risk of abnormal hostility, a 9.5% higher risk of abnormal phobic anxiety, a 10.4% higher risk of abnormal paranoid ideation, and an 11.0% higher risk of abnormal psychoticism. Extensive documentation exists on the physical health implications of obesity, indicating a consistent association between elevated BMI and heightened risks of chronic diseases and mortality [28–30]. Nevertheless, a growing body of research examining the psychological effects of obesity produces inconsistent results. Most studies indicate an inverse correlation between body weight and psychological well-being [31–34]. However, some studies suggest a positive association instead [35], while others find either a neutral or insignificant correlation [36]. In our study, we provided a more precise depiction of the association of BMI with individuals’ mental well-being. Engaging in regular exercise and making dietary changes to lower BMI could potentially alleviate somatic symptoms linked to psychological distress. Those with lower BMI typically report fewer physical complaints and demonstrate enhanced coping mechanisms for stressors.

Our research revealed that for every one-point increase in lung capacity, the risk of abnormal somatization decreases by 1.8%, the risk of abnormal obsessive-compulsive disorder decreases by 2.0%, the risk of abnormal interpersonal sensitivity decreases by 1.6%, the risk of abnormal depression decreases by 5.6%, the risk of abnormal anxiety decreases by 1.9%, the risk of abnormal hostility decreases by 1.5%, the risk of abnormal paranoid ideation decreases by 1.4%, and the risk of abnormal psychoticism decreases by 1.9%. Increasing evidence in current research suggests a close association between obstructive pulmonary diseases such as asthma, chronic bronchitis, and emphysema, and psychological health issues like depression and anxiety [37–41]. Previous research conducted among adult clinical and general practice populations has revealed elevated rates of anxiety and mood disorders, particularly major depression [42–47]. Community-based studies have confirmed and expanded upon the general validity of the association between asthma, chronic obstructive pulmonary disease, and mental disorders [39, 48–54]. Our study aligns with previous research but provides a more nuanced examination of the association between lung capacity and psychological well-being.

Our study indicates that for every one-point increase in endurance running, the risk of abnormal somatization decreases by 2.7%, the risk of abnormal obsessive-compulsive disorder decreases by 3.0%, the risk of abnormal interpersonal sensitivity decreases by 3.1%, the risk of abnormal depression decreases by 16.5%, the risk of abnormal anxiety decreases by 1.7%, the risk of abnormal hostility decreases by 2.3%, the risk of abnormal phobic anxiety decreases by 1.7%, the risk of abnormal paranoid ideation decreases by 1.9%, and the risk of abnormal psychoticism decreases by 2.0%. Additionally, we found that for every point increase in lower limb explosive force, there is a 2.0% increase in the risk of abnormal anxiety among students, indicating an adverse effect on mental health. There is considerable evidence substantiating the link between physical activity and different mental health results throughout all stages of life [55–57]. Long-term running interventions frequently enhance mental health metrics, particularly depression indicators, among individuals with psychosis [58–66]. Some prior research suggests that running enhances mood, especially when conducted outdoors, across various intensities, except for an intensity significantly above the lactate threshold [67–69]. Our study supplements previous research by emphasizing the impact of endurance running scores on psychological resilience and validating the significant role of endurance running in various psychological indicators, with particular effectiveness observed in depression indicators.

The study delves into the complex association between physical fitness and mental health, revealing a potential correlation between these two domains. It demonstrates the substantial impact of interventions aimed at lowering BMI, enhancing lung capacity, and engaging in endurance running on various facets of mental well-being, including somatization, obsessive tendencies, interpersonal sensitivity, depression, anxiety, hostility, phobic anxiety, paranoid ideation, and psychosis. These findings underscore the pivotal role of physical activity in promoting mental health. This potential correlation between physical fitness and mental health can be elucidated through various underlying mechanisms [70]. Firstly, interventions targeting BMI reduction through regular exercise and dietary adjustments show promise in alleviating somatic symptoms associated with psychological distress. These effects may be attributed to exercise-induced physiological changes, such as improved cardiovascular health and hormonal regulation, which positively impact mood and emotional well-being. Similarly, enhancements in lung capacity and engagement in endurance running have been associated with reduced obsessive-compulsive tendencies, possibly mediated by neurotransmitter modulation in the brain. Furthermore, the release of endorphins and serotonin during exercise may further contribute to the amelioration of depressive and anxious symptoms, thereby enhancing mental well-being. Moreover, endurance running has been shown to facilitate emotional regulation and empathy by fostering social bonding and communication skills, thereby enhancing interpersonal sensitivity. Physical activity also serves as a constructive outlet for managing stress and aggression, leading to decreased hostility and improved emotional well-being. Additionally, interventions aimed at enhancing lung capacity and engaging in endurance running may mitigate symptoms of phobic-anxiety disorders by promoting relaxation and stress relief. Furthermore, strategies targeting BMI reduction and regular physical activity maintenance can contribute to reduced paranoid ideation by bolstering self-esteem and self-efficacy.

In summary, these findings offer valuable insights into strategies for enhancing overall health and well-being, emphasizing the importance of integrating physical activity into mental health management approaches. By understanding the potential correlation between physical fitness and mental health and implementing appropriate interventions, individuals can take proactive steps towards improving their mental well-being and achieving a better quality of life.

### Limitations

While our study offers valuable insights into the correlation between physical fitness and mental health, it is not without limitations. The cross-sectional design limits our ability to determine causality, and selection bias may arise since participants likely have higher physical activity levels than the general population, potentially skewing mental health outcomes positively. Additionally, the use of self-reported mental health measures might introduce reporting bias, affecting the accuracy of associations. The generalizability of our findings could also be influenced by the demographic and geographic characteristics of our sample. Furthermore, this study did not employ multilevel modeling, despite the hierarchical nature of the data, due to complexity and sample size constraints, which might limit the added value of this approach for our specific research questions. Addressing these biases and limitations in future longitudinal studies, and considering multilevel models, could strengthen the validity of our findings and enable a more comprehensive interpretation of interactions across different levels of data. Future research involving diverse populations across various settings is essential to validate and expand our conclusions on a global scale.

## Conclusion

In summary, lowering BMI, increasing lung capacity, and improving endurance running have shown promising benefits for various dimensions of mental health. Incorporating regular physical activity into lifestyle interventions may serve as an effective strategy for promoting holistic well-being and reducing the burden of mental health disorders. Further research is warranted to explore the mechanisms underlying these associations and to develop targeted interventions for improving mental health outcomes through physical fitness interventions.

### Electronic supplementary material

Below is the link to the electronic supplementary material.

Supplementary Material 1

Supplementary Material 2

## Data Availability

No datasets were generated or analysed during the current study.

## Declarations

### Patient and public involvement

Patients and/or the public were not involved in the design, or conduct, or reporting, or dissemination plans of this research.

### Provenance and peer review

Not commissioned; externally peer reviewed.
