# Supplementary material for: The effect of physical fitness on psychological health: evidence from Chinese university students
Source: BMC Public Health. 2024 May 21;24:1365. doi: 10.1186/s12889-024-18841-y (PMC11106851; doi:10.1186/s12889-024-18841-y)
Supplement: Supplementary file 2 — Supplementary Material 2 [file 12889_2024_18841_MOESM2_ESM.doc]

**National Standards of Student Physical Fitness and Health (Revised in 2014)**

**I. Introduction**

1. The "National Student Physical Fitness and Health Standards" (hereinafter referred to as "the Standards") serve as a fundamental guiding document for national school education work and a basic criterion for educational quality. They are crucial for evaluating students' comprehensive qualities, assessing school performance, and measuring educational development across various regions. These standards represent the specific implementation of the "National Physical Exercise Standards" in schools and are applicable to students in full-time regular elementary schools, middle schools, high schools, secondary vocational schools, and regular institutions of higher education.

2. The revision of these standards adheres to the principle of health first. It implements the requirements of the "National Mid- and Long-term Education Reform and Development Plan Outline (2010-2020)", the "Notice of the General Office of the State Council Forwarding the Opinions of the Ministry of Education and Other Departments on Further Strengthening School Physical Education Work" (State Office Issuance [2012] No. 53), and the "Notice of the Ministry of Education on Issuing the 'Student Physical Fitness and Health Monitoring and Evaluation Methods' and Other Documents" (Ministry of Education, Sports and Arts [2014] No. 3). The focus is on enhancing the reliability, validity, and discriminability of the "Standards", strengthening their role in educational motivation, feedback adjustment, and guiding physical exercises, and improving their support capability in educational monitoring and performance evaluation.

3. These standards comprehensively assess students' physical health levels from aspects of physical form, physical function, and physical fitness. They serve as educational tools to promote students' physical development, encourage active participation in physical exercises, and are a vital component of the national student development core competency system and academic quality standards, representing individual evaluation criteria for student physical health.

4. The standards categorize applicable subjects into the following groups: each grade in elementary, middle, and high school is considered as one group, with six groups in elementary school, three groups in middle school, and three groups in high school. In universities, the first and second years are grouped together, and the third and fourth years are another group.

5. For elementary, middle, high schools, and universities, the test indicators for each group are mandatory. These include height and weight under the category of physical form, lung capacity under physical function, and the 50-meter run and sit-and-reach under physical fitness, which are common indicators for students across all grades.

6. The annual total score of these standards consists of the sum of the standard score and additional points, with a full score being 120 points. The standard score is the sum of the scores of each individual indicator multiplied by its weight, with a full score of 100 points. Additional points are determined based on actual performance, meaning points are added for indicators where the score exceeds 100 points, up to a maximum of 20 points. For elementary schools, the additional indicator is the 1-minute rope skipping with a bonus of up to 20 points. For middle schools, high schools, and universities, the additional indicators include pull-ups for boys and a 1000-meter run, and a 1-minute sit-up and 800-meter run for girls, with each indicator offering a bonus of up to 10 points.

7. Students' annual total scores determine their grades: 90.0 points and above is excellent, 80.0 to 89.9 points is good, 60.0 to 79.9 points is passing, and 59.9 points and below is failing.

8. Each student is evaluated annually and their score is recorded in the "National Student Physical Fitness and Health Standards Registration Card" (Appendices 1-6). Schools with special education systems may add or remove columns in the registration card as per regulations and needs. Students' final grades and levels upon graduation are determined by combining 50% of the score from the graduation year's total annual score and 50% of the average total annual score from other years.

9. Students who achieve a good or higher rating in the test are eligible for commendation and awards; those who achieve an excellent rating can earn physical education scholarship credits. Students who fail the test are allowed one retest within the academic year. If they fail the retest, their annual score is recorded as failing. For students in regular high schools, secondary vocational schools, and regular higher education institutions, those who score below 50 points in the "Standards" test at graduation are subject to completion or dropout procedures.

10. Students with illnesses or disabilities may submit a request to the school for a temporary postponement or exemption from the "Standards". Upon verification by a medical institution and approval by the physical education department, the execution of the "Standards" can be postponed or exempted. This should be documented in the "Exemption Application Form for National Student Physical Fitness and Health Standards" (Appendix 7) and filed in the student's record. Students with disabilities who are exempted from the "Standards" due to a genuine loss of physical ability can still participate in commendation and awards, and their exemption status should be noted in the "Standards" grade upon graduation.

11. Each school conducts the "Standards" testing annually for students of all grades. After the testing data is reviewed and approved by the local educational administrative department as required, it is uploaded to the "National Student Physical Fitness and Health Standards Data Management System" via the "China Student Physical Fitness and Health Network". The timing for testing and data uploading is determined by the educational administrative department.

12. The interpretation of these standards is the responsibility of the Ministry of Education.

**II. Individual Indicators and Their Weights**

| Test Subjects | Individual Indicators | Weight (%) |
| --- | --- | --- |
| From first grade in elementary school to fourth year in university | Body Mass Index (BMI) | 15 |
|  | Lung Capacity | 15 |
| First and second grades in elementary school | 50-meter Run | 20 |
|  | Sit-and-Reach | 30 |
|  | 1-minute Rope Skipping | 20 |
| Third and fourth grades in elementary school | 50-meter Run | 20 |
|  | Sit-and-Reach | 20 |
|  | 1-minute Rope Skipping | 20 |
|  | 1-minute Sit-up | 10 |
| Fifth and sixth grades in elementary school | 50-meter Run | 20 |
|  | Sit-and-Reach | 10 |
|  | 1-minute Rope Skipping | 10 |
|  | 1-minute Sit-up | 20 |
|  | 50-meter × 8 Shuttle Run | 10 |
| Each grade in middle school, high school, and university | 50-meter Run | 20 |
|  | Sit-and-Reach | 10 |
|  | Standing Long Jump | 10 |
|  | Pull-ups (boys) / 1-minute Sit-up (girls) | 10 |
|  | 1000-meter Run (boys) / 800-meter Run (girls) | 20 |

Note: Body Mass Index (BMI) = Weight (kg) / Height^2^ (m^2^).

**III. Scoring Tables**

(I) Individual Indicator Scoring Tables

**Table 1-1: BMI Scoring Table for Boys (Unit: kg/m^2^)**

| **Level** | **Individual Score** | **Grade 1** | **Grade 2** | **Grade 3** | **Grade 4** | **Grade 5** | **Grade 6** | **Junior 1** | **Junior 2** | **Junior 3** | **Senior 1** | **Senior 2** | **Senior 3** | **University** |
| --- | --- | --- | --- | --- | --- | --- | --- | --- | --- | --- | --- | --- | --- | --- |
| **Normal** | **100** | 13.5~18.1 | 13.7~18.4 | 13.9~19.4 | 14.2~20.1 | 14.4~21.4 | 14.7~21.8 | 15.5~22.1 | 15.7~22.5 | 15.8~22.8 | 16.5~23.2 | 16.8~23.7 | 17.3~23.8 | 17.9~23.9 |
| **Underweight** | **80** | ≤13.4 | ≤13.6 | ≤13.8 | ≤14.1 | ≤14.3 | ≤14.6 | ≤15.4 | ≤15.6 | ≤15.7 | ≤16.4 | ≤16.7 | ≤17.2 | ≤17.8 |
| **Overweight** |  | 18.2~20.3 | 18.5~20.4 | 19.5~22.1 | 20.2~22.6 | 21.5~24.1 | 21.9~24.5 | 22.2~24.9 | 22.6~25.2 | 22.9~26.0 | 23.3~26.3 | 23.8~26.5 | 23.9~27.3 | 24.0~27.9 |
| **Obese** | **60** | ≥20.4 | ≥20.5 | ≥22.2 | ≥22.7 | ≥24.2 | ≥24.6 | ≥25.0 | ≥25.3 | ≥26.1 | ≥26.4 | ≥26.6 | ≥27.4 | ≥28.0 |

**Table 1-2: BMI Scoring Table for Girls (Unit: kg/m^2^)**

| **Level** | **Individual Score** | **Grade 1** | **Grade 2** | **Grade 3** | **Grade 4** | **Grade 5** | **Grade 6** | **Junior 1** | **Junior 2** | **Junior 3** | **Senior 1** | **Senior 2** | **Senior 3** | **University** |
| --- | --- | --- | --- | --- | --- | --- | --- | --- | --- | --- | --- | --- | --- | --- |
| **Normal** | **100** | 13.3~17.3 | 13.5~17.8 | 13.6~18.6 | 13.7~19.4 | 13.8~20.5 | 14.2~20.8 | 14.8~21.7 | 15.3~22.2 | 16.0~22.6 | 16.5~22.7 | 16.9~23.2 | 17.1~23.3 | 17.2~23.9 |
| **Underweight** | **80** | ≤13.2 | ≤13.4 | ≤13.5 | ≤13.6 | ≤13.7 | ≤14.1 | ≤14.7 | ≤15.2 | ≤15.9 | ≤16.4 | ≤16.8 | ≤17.0 | ≤17.1 |
| **Overweight** |  | 17.4~19.2 | 17.9~20.2 | 18.7~21.1 | 19.5~22.0 | 20.6~22.9 | 20.9~23.6 | 21.8~24.4 | 22.3~24.8 | 22.7~25.1 | 22.8~25.2 | 23.3~25.4 | 23.4~25.7 | 24.0~27.9 |
| **Obese** | **60** | ≥19.3 | ≥20.3 | ≥21.2 | ≥22.1 | ≥23.0 | ≥23.7 | ≥24.5 | ≥24.9 | ≥25.2 | ≥25.3 | ≥25.5 | ≥25.8 | ≥28.0 |

**Table 1-3: Lung Capacity Scoring Table for Boys (Unit: ml)**

| **Level** | **Individual Score** | **Grade 1** | **Grade 2** | **Grade 3** | **Grade 4** | **Grade 5** | **Grade 6** | **Junior 1** | **Junior 2** | **Junior 3** | **Senior 1** | **Senior 2** | **Senior 3** | **Freshman**  **Sophomore** | **Junior**  **Senior** |
| --- | --- | --- | --- | --- | --- | --- | --- | --- | --- | --- | --- | --- | --- | --- | --- |
| **Excellent** | **100** | 1700 | 2000 | 2300 | 2600 | 2900 | 3200 | 3640 | 3940 | 4240 | 4540 | 4740 | 4940 | 5040 | 5140 |
|  | **95** | 1600 | 1900 | 2200 | 2500 | 2800 | 3100 | 3520 | 3820 | 4120 | 4420 | 4620 | 4820 | 4920 | 5020 |
|  | **90** | 1500 | 1800 | 2100 | 2400 | 2700 | 3000 | 3400 | 3700 | 4000 | 4300 | 4500 | 4700 | 4800 | 4900 |
| **Good** | **85** | 1400 | 1650 | 1900 | 2150 | 2450 | 2750 | 3150 | 3450 | 3750 | 4050 | 4250 | 4450 | 4550 | 4650 |
|  | **80** | 1300 | 1500 | 1700 | 1900 | 2200 | 2500 | 2900 | 3200 | 3500 | 3800 | 4000 | 4200 | 4300 | 4400 |
| **Pass** | **78** | 1240 | 1430 | 1620 | 1820 | 2110 | 2400 | 2780 | 3080 | 3380 | 3680 | 3880 | 4080 | 4180 | 4280 |
|  | **76** | 1180 | 1360 | 1540 | 1740 | 2020 | 2300 | 2660 | 2960 | 3260 | 3560 | 3760 | 3960 | 4060 | 4160 |
|  | **74** | 1120 | 1290 | 1460 | 1660 | 1930 | 2200 | 2540 | 2840 | 3140 | 3440 | 3640 | 3840 | 3940 | 4040 |
|  | **72** | 1060 | 1220 | 1380 | 1580 | 1840 | 2100 | 2420 | 2720 | 3020 | 3320 | 3520 | 3720 | 3820 | 3920 |
|  | **70** | 1000 | 1150 | 1300 | 1500 | 1750 | 2000 | 2300 | 2600 | 2900 | 3200 | 3400 | 3600 | 3700 | 3800 |
|  | **68** | 940 | 1080 | 1220 | 1420 | 1660 | 1900 | 2180 | 2480 | 2780 | 3080 | 3280 | 3480 | 3580 | 3680 |
|  | **66** | 880 | 1010 | 1140 | 1340 | 1570 | 1800 | 2060 | 2360 | 2660 | 2960 | 3160 | 3360 | 3460 | 3560 |
|  | **64** | 820 | 940 | 1060 | 1260 | 1480 | 1700 | 1940 | 2240 | 2540 | 2840 | 3040 | 3240 | 3340 | 3440 |
|  | **62** | 760 | 870 | 980 | 1180 | 1390 | 1600 | 1820 | 2120 | 2420 | 2720 | 2920 | 3120 | 3220 | 3320 |
|  | **60** | 700 | 800 | 900 | 1100 | 1300 | 1500 | 1700 | 2000 | 2300 | 2600 | 2800 | 3000 | 3100 | 3200 |
| **Fail** | **50** | 660 | 750 | 840 | 1030 | 1220 | 1410 | 1600 | 1890 | 2180 | 2470 | 2660 | 2850 | 2940 | 3030 |
|  | **40** | 620 | 700 | 780 | 960 | 1140 | 1320 | 1500 | 1780 | 2060 | 2340 | 2520 | 2700 | 2780 | 2860 |
|  | **30** | 580 | 650 | 720 | 890 | 1060 | 1230 | 1400 | 1670 | 1940 | 2210 | 2380 | 2550 | 2620 | 2690 |
|  | **20** | 540 | 600 | 660 | 820 | 980 | 1140 | 1300 | 1560 | 1820 | 2080 | 2240 | 2400 | 2460 | 2520 |
|  | **10** | 500 | 550 | 600 | 750 | 900 | 1050 | 1200 | 1450 | 1700 | 1950 | 2100 | 2250 | 2300 | 2350 |

**Table 1-4: Lung Capacity Scoring Table for Girls (Unit: ml)**

| **Level** | **Individual Score** | **Grade 1** | **Grade 2** | **Grade 3** | **Grade 4** | **Grade 5** | **Grade 6** | **Junior 1** | **Junior 2** | **Junior 3** | **Senior 1** | **Senior 2** | **Senior 3** | **Freshman**  **Sophomore** | **Junior**  **Senior** |
| --- | --- | --- | --- | --- | --- | --- | --- | --- | --- | --- | --- | --- | --- | --- | --- |
| **Excellent** | **100** | 1400 | 1600 | 1800 | 2000 | 2250 | 2500 | 2750 | 2900 | 3050 | 3150 | 3250 | 3350 | 3400 | 3450 |
|  | **95** | 1300 | 1500 | 1700 | 1900 | 2150 | 2400 | 2650 | 2850 | 3000 | 3100 | 3200 | 3300 | 3350 | 3400 |
|  | **90** | 1200 | 1400 | 1600 | 1800 | 2050 | 2300 | 2550 | 2800 | 2950 | 3050 | 3150 | 3250 | 3300 | 3350 |
| **Good** | **85** | 1100 | 1300 | 1500 | 1700 | 1950 | 2200 | 2450 | 2650 | 2800 | 2900 | 3000 | 3100 | 3150 | 3200 |
|  | **80** | 1000 | 1200 | 1400 | 1600 | 1850 | 2100 | 2350 | 2500 | 2650 | 2750 | 2850 | 2950 | 3000 | 3050 |
| **Pass** | **78** | 960 | 1150 | 1340 | 1530 | 1770 | 2010 | 2250 | 2400 | 2550 | 2650 | 2750 | 2850 | 2900 | 2950 |
|  | **76** | 920 | 1100 | 1280 | 1460 | 1690 | 1920 | 2150 | 2300 | 2450 | 2550 | 2650 | 2750 | 2800 | 2850 |
|  | **74** | 880 | 1050 | 1220 | 1390 | 1610 | 1830 | 2050 | 2200 | 2350 | 2450 | 2550 | 2650 | 2700 | 2750 |
|  | **72** | 840 | 1000 | 1160 | 1320 | 1530 | 1740 | 1950 | 2100 | 2250 | 2350 | 2450 | 2550 | 2600 | 2650 |
|  | **70** | 800 | 950 | 1100 | 1250 | 1450 | 1650 | 1850 | 2000 | 2150 | 2250 | 2350 | 2450 | 2500 | 2550 |
|  | **68** | 760 | 900 | 1040 | 1180 | 1370 | 1560 | 1750 | 1900 | 2050 | 2150 | 2250 | 2350 | 2400 | 2450 |
|  | **66** | 720 | 850 | 980 | 1110 | 1290 | 1470 | 1650 | 1800 | 1950 | 2050 | 2150 | 2250 | 2300 | 2350 |
|  | **64** | 680 | 800 | 920 | 1040 | 1210 | 1380 | 1550 | 1700 | 1850 | 1950 | 2050 | 2150 | 2200 | 2250 |
|  | **62** | 640 | 750 | 860 | 970 | 1130 | 1290 | 1450 | 1600 | 1750 | 1850 | 1950 | 2050 | 2100 | 2150 |
|  | **60** | 600 | 700 | 800 | 900 | 1050 | 1200 | 1350 | 1500 | 1650 | 1750 | 1850 | 1950 | 2000 | 2050 |
| **Fail** | **50** | 580 | 680 | 780 | 880 | 1020 | 1170 | 1310 | 1460 | 1610 | 1710 | 1810 | 1910 | 1960 | 2010 |
|  | **40** | 560 | 660 | 760 | 860 | 990 | 1140 | 1270 | 1420 | 1570 | 1670 | 1770 | 1870 | 1920 | 1970 |
|  | **30** | 540 | 640 | 740 | 840 | 960 | 1110 | 1230 | 1380 | 1530 | 1630 | 1730 | 1830 | 1880 | 1930 |
|  | **20** | 520 | 620 | 720 | 820 | 930 | 1080 | 1190 | 1340 | 1490 | 1590 | 1690 | 1790 | 1840 | 1890 |
|  | **10** | 500 | 600 | 700 | 800 | 900 | 1050 | 1150 | 1300 | 1450 | 1550 | 1650 | 1750 | 1800 | 1850 |

**Table 1-5: 50-meter Run Scoring Table for Boys (Unit: seconds)**

| **Level** | **Individual Score** | **Grade 1** | **Grade 2** | **Grade 3** | **Grade 4** | **Grade 5** | **Grade 6** | **Junior 1** | **Junior 2** | **Junior 3** | **Senior 1** | **Senior 2** | **Senior 3** | **Freshman**  **Sophomore** | **Junior**  **Senior** |
| --- | --- | --- | --- | --- | --- | --- | --- | --- | --- | --- | --- | --- | --- | --- | --- |
| **Excellent** | **100** | 10.2 | 9.6 | 9.1 | 8.7 | 8.4 | 8.2 | 7.8 | 7.5 | 7.3 | 7.1 | 7.0 | 6.8 | 6.7 | 6.6 |
|  | **95** | 10.3 | 9.7 | 9.2 | 8.8 | 8.5 | 8.3 | 7.9 | 7.6 | 7.4 | 7.2 | 7.1 | 6.9 | 6.8 | 6.7 |
|  | **90** | 10.4 | 9.8 | 9.3 | 8.9 | 8.6 | 8.4 | 8.0 | 7.7 | 7.5 | 7.3 | 7.2 | 7.0 | 6.9 | 6.8 |
| **Good** | **85** | 10.5 | 9.9 | 9.4 | 9.0 | 8.7 | 8.5 | 8.1 | 7.8 | 7.6 | 7.4 | 7.3 | 7.1 | 7.0 | 6.9 |
|  | **80** | 10.6 | 10.0 | 9.5 | 9.1 | 8.8 | 8.6 | 8.2 | 7.9 | 7.7 | 7.5 | 7.4 | 7.2 | 7.1 | 7.0 |
| **Pass** | **78** | 10.8 | 10.2 | 9.7 | 9.3 | 9.0 | 8.8 | 8.4 | 8.1 | 7.9 | 7.7 | 7.6 | 7.4 | 7.3 | 7.2 |
|  | **76** | 11.0 | 10.4 | 9.9 | 9.5 | 9.2 | 9.0 | 8.6 | 8.3 | 8.1 | 7.9 | 7.8 | 7.6 | 7.5 | 7.4 |
|  | **74** | 11.2 | 10.6 | 10.1 | 9.7 | 9.4 | 9.2 | 8.8 | 8.5 | 8.3 | 8.1 | 8.0 | 7.8 | 7.7 | 7.6 |
|  | **72** | 11.4 | 10.8 | 10.3 | 9.9 | 9.6 | 9.4 | 9.0 | 8.7 | 8.5 | 8.3 | 8.2 | 8.0 | 7.9 | 7.8 |
|  | **70** | 11.6 | 11.0 | 10.5 | 10.1 | 9.8 | 9.6 | 9.2 | 8.9 | 8.7 | 8.5 | 8.4 | 8.2 | 8.1 | 8.0 |
|  | **68** | 11.8 | 11.2 | 10.7 | 10.3 | 10.0 | 9.8 | 9.4 | 9.1 | 8.9 | 8.7 | 8.6 | 8.4 | 8.3 | 8.2 |
|  | **66** | 12.0 | 11.4 | 10.9 | 10.5 | 10.2 | 10.0 | 9.6 | 9.3 | 9.1 | 8.9 | 8.8 | 8.6 | 8.5 | 8.4 |
|  | **64** | 12.2 | 11.6 | 11.1 | 10.7 | 10.4 | 10.2 | 9.8 | 9.5 | 9.3 | 9.1 | 9.0 | 8.8 | 8.7 | 8.6 |
|  | **62** | 12.4 | 11.8 | 11.3 | 10.9 | 10.6 | 10.4 | 10.0 | 9.7 | 9.5 | 9.3 | 9.2 | 9.0 | 8.9 | 8.8 |
|  | **60** | 12.6 | 12.0 | 11.5 | 11.1 | 10.8 | 10.6 | 10.2 | 9.9 | 9.7 | 9.5 | 9.4 | 9.2 | 9.1 | 9.0 |
| **Fail** | **50** | 12.8 | 12.2 | 11.7 | 11.3 | 11.0 | 10.8 | 10.4 | 10.1 | 9.9 | 9.7 | 9.6 | 9.4 | 9.3 | 9.2 |
|  | **40** | 13.0 | 12.4 | 11.9 | 11.5 | 11.2 | 11.0 | 10.6 | 10.3 | 10.1 | 9.9 | 9.8 | 9.6 | 9.5 | 9.4 |
|  | **30** | 13.2 | 12.6 | 12.1 | 11.7 | 11.4 | 11.2 | 10.8 | 10.5 | 10.3 | 10.1 | 10.0 | 9.8 | 9.7 | 9.6 |
|  | **20** | 13.4 | 12.8 | 12.3 | 11.9 | 11.6 | 11.4 | 11.0 | 10.7 | 10.5 | 10.3 | 10.2 | 10.0 | 9.9 | 9.8 |
|  | **10** | 13.6 | 13.0 | 12.5 | 12.1 | 11.8 | 11.6 | 11.2 | 10.9 | 10.7 | 10.5 | 10.4 | 10.2 | 10.1 | 10.0 |

**Table 1-6: 50-meter Run Scoring Table for Girls (Unit: seconds)**

| **Level** | **Individual Score** | **Grade 1** | **Grade 2** | **Grade 3** | **Grade 4** | **Grade 5** | **Grade 6** | **Junior 1** | **Junior 2** | **Junior 3** | **Senior 1** | **Senior 2** | **Senior 3** | **Freshman**  **Sophomore** | **Junior**  **Senior** |
| --- | --- | --- | --- | --- | --- | --- | --- | --- | --- | --- | --- | --- | --- | --- | --- |
| **Excellent** | **100** | 11.0 | 10.0 | 9.2 | 8.7 | 8.3 | 8.2 | 8.1 | 8.0 | 7.9 | 7.8 | 7.7 | 7.6 | 7.5 | 7.4 |
|  | **95** | 11.1 | 10.1 | 9.3 | 8.8 | 8.4 | 8.3 | 8.2 | 8.1 | 8.0 | 7.9 | 7.8 | 7.7 | 7.6 | 7.5 |
|  | **90** | 11.2 | 10.2 | 9.4 | 8.9 | 8.5 | 8.4 | 8.3 | 8.2 | 8.1 | 8.0 | 7.9 | 7.8 | 7.7 | 7.6 |
| **Good** | **85** | 11.5 | 10.5 | 9.7 | 9.2 | 8.8 | 8.7 | 8.6 | 8.5 | 8.4 | 8.3 | 8.2 | 8.1 | 8.0 | 7.9 |
|  | **80** | 11.8 | 10.8 | 10.0 | 9.5 | 9.1 | 9.0 | 8.9 | 8.8 | 8.7 | 8.6 | 8.5 | 8.4 | 8.3 | 8.2 |
| **Pass** | **78** | 12.0 | 11.0 | 10.2 | 9.7 | 9.3 | 9.2 | 9.1 | 9.0 | 8.9 | 8.8 | 8.7 | 8.6 | 8.5 | 8.4 |
|  | **76** | 12.2 | 11.2 | 10.4 | 9.9 | 9.5 | 9.4 | 9.3 | 9.2 | 9.1 | 9.0 | 8.9 | 8.8 | 8.7 | 8.6 |
|  | **74** | 12.4 | 11.4 | 10.6 | 10.1 | 9.7 | 9.6 | 9.5 | 9.4 | 9.3 | 9.2 | 9.1 | 9.0 | 8.9 | 8.8 |
|  | **72** | 12.6 | 11.6 | 10.8 | 10.3 | 9.9 | 9.8 | 9.7 | 9.6 | 9.5 | 9.4 | 9.3 | 9.2 | 9.1 | 9.0 |
|  | **70** | 12.8 | 11.8 | 11.0 | 10.5 | 10.1 | 10.0 | 9.9 | 9.8 | 9.7 | 9.6 | 9.5 | 9.4 | 9.3 | 9.2 |
|  | **68** | 13.0 | 12.0 | 11.2 | 10.7 | 10.3 | 10.2 | 10.1 | 10.0 | 9.9 | 9.8 | 9.7 | 9.6 | 9.5 | 9.4 |
|  | **66** | 13.2 | 12.2 | 11.4 | 10.9 | 10.5 | 10.4 | 10.3 | 10.2 | 10.1 | 10.0 | 9.9 | 9.8 | 9.7 | 9.6 |
|  | **64** | 13.4 | 12.4 | 11.6 | 11.1 | 10.7 | 10.6 | 10.5 | 10.4 | 10.3 | 10.2 | 10.1 | 10.0 | 9.9 | 9.8 |
|  | **62** | 13.6 | 12.6 | 11.8 | 11.3 | 10.9 | 10.8 | 10.7 | 10.6 | 10.5 | 10.4 | 10.3 | 10.2 | 10.1 | 10.0 |
|  | **60** | 13.8 | 12.8 | 12.0 | 11.5 | 11.1 | 11.0 | 10.9 | 10.8 | 10.7 | 10.6 | 10.5 | 10.4 | 10.3 | 10.2 |
| **Fail** | **50** | 14.0 | 13.0 | 12.2 | 11.7 | 11.3 | 11.2 | 11.1 | 11.0 | 10.9 | 10.8 | 10.7 | 10.6 | 10.5 | 10.4 |
|  | **40** | 14.2 | 13.2 | 12.4 | 11.9 | 11.5 | 11.4 | 11.3 | 11.2 | 11.1 | 11.0 | 10.9 | 10.8 | 10.7 | 10.6 |
|  | **30** | 14.4 | 13.4 | 12.6 | 12.1 | 11.7 | 11.6 | 11.5 | 11.4 | 11.3 | 11.2 | 11.1 | 11.0 | 10.9 | 10.8 |
|  | **20** | 14.6 | 13.6 | 12.8 | 12.3 | 11.9 | 11.8 | 11.7 | 11.6 | 11.5 | 11.4 | 11.3 | 11.2 | 11.1 | 11.0 |
|  | **10** | 14.8 | 13.8 | 13.0 | 12.5 | 12.1 | 12.0 | 11.9 | 11.8 | 11.7 | 11.6 | 11.5 | 11.4 | 11.3 | 11.2 |

**Table 1-7: Sit-and-Reach Scoring Table for Boys (Unit: cm)**

| **Level** | **Individual Score** | **Grade 1** | **Grade 2** | **Grade 3** | **Grade 4** | **Grade 5** | **Grade 6** | **Junior 1** | **Junior 2** | **Junior 3** | **Senior 1** | **Senior 2** | **Senior 3** | **Freshman**  **Sophomore** | **Junior**  **Senior** |
| --- | --- | --- | --- | --- | --- | --- | --- | --- | --- | --- | --- | --- | --- | --- | --- |
| **Excellent** | **100** | 16.1 | 16.2 | 16.3 | 16.4 | 16.5 | 16.6 | 17.6 | 19.6 | 21.6 | 23.6 | 24.3 | 24.6 | 24.9 | 25.1 |
|  | **95** | 14.6 | 14.7 | 14.9 | 15.0 | 15.2 | 15.3 | 15.9 | 17.7 | 19.7 | 21.5 | 22.4 | 22.8 | 23.1 | 23.3 |
|  | **90** | 13.0 | 13.2 | 13.4 | 13.6 | 13.8 | 14.0 | 14.2 | 15.8 | 17.8 | 19.4 | 20.5 | 21.0 | 21.3 | 21.5 |
| **Good** | **85** | 12.0 | 11.9 | 11.8 | 11.7 | 11.6 | 11.5 | 12.3 | 13.7 | 15.8 | 17.2 | 18.3 | 19.1 | 19.5 | 19.9 |
|  | **80** | 11.0 | 10.6 | 10.2 | 9.8 | 9.4 | 9.0 | 10.4 | 11.6 | 13.8 | 15.0 | 16.1 | 17.2 | 17.7 | 18.2 |
| **Pass** | **78** | 9.9 | 9.5 | 9.1 | 8.6 | 8.2 | 7.7 | 9.1 | 10.3 | 12.4 | 13.6 | 14.7 | 15.8 | 16.3 | 16.8 |
|  | **76** | 8.8 | 8.4 | 8.0 | 7.4 | 7.0 | 6.4 | 7.8 | 9.0 | 11.0 | 12.2 | 13.3 | 14.4 | 14.9 | 15.4 |
|  | **74** | 7.7 | 7.3 | 6.9 | 6.2 | 5.8 | 5.1 | 6.5 | 7.7 | 9.6 | 10.8 | 11.9 | 13.0 | 13.5 | 14.0 |
|  | **72** | 6.6 | 6.2 | 5.8 | 5.0 | 4.6 | 3.8 | 5.2 | 6.4 | 8.2 | 9.4 | 10.5 | 11.6 | 12.1 | 12.6 |
|  | **70** | 5.5 | 5.1 | 4.7 | 3.8 | 3.4 | 2.5 | 3.9 | 5.1 | 6.8 | 8.0 | 9.1 | 10.2 | 10.7 | 11.2 |
|  | **68** | 4.4 | 4.0 | 3.6 | 2.6 | 2.2 | 1.2 | 2.6 | 3.8 | 5.4 | 6.6 | 7.7 | 8.8 | 9.3 | 9.8 |
|  | **66** | 3.3 | 2.9 | 2.5 | 1.4 | 1.0 | -0.1 | 1.3 | 2.5 | 4.0 | 5.2 | 6.3 | 7.4 | 7.9 | 8.4 |
|  | **64** | 2.2 | 1.8 | 1.4 | 0.2 | -0.2 | -1.4 | 0.0 | 1.2 | 2.6 | 3.8 | 4.9 | 6.0 | 6.5 | 7.0 |
|  | **62** | 1.1 | 0.7 | 0.3 | -1.0 | -1.4 | -2.7 | -1.3 | -0.1 | 1.2 | 2.4 | 3.5 | 4.6 | 5.1 | 5.6 |
|  | **60** | 0.0 | -0.4 | -0.8 | -2.2 | -2.6 | -4.0 | -2.6 | -1.4 | -0.2 | 1.0 | 2.1 | 3.2 | 3.7 | 4.2 |
| **Fail** | **50** | -0.8 | -1.2 | -1.6 | -3.2 | -3.6 | -5.0 | -3.8 | -2.6 | -1.4 | 0.0 | 1.1 | 2.2 | 2.7 | 3.2 |
|  | **40** | -1.6 | -2.0 | -2.4 | -4.2 | -4.6 | -6.0 | -5.0 | -3.8 | -2.6 | -1.0 | 0.1 | 1.2 | 1.7 | 2.2 |
|  | **30** | -2.4 | -2.8 | -3.2 | -5.2 | -5.6 | -7.0 | -6.2 | -5.0 | -3.8 | -2.0 | -0.9 | 0.2 | 0.7 | 1.2 |
|  | **20** | -3.2 | -3.6 | -4.0 | -6.2 | -6.6 | -8.0 | -7.4 | -6.2 | -5.0 | -3.0 | -1.9 | -0.8 | -0.3 | 0.2 |
|  | **10** | -4.0 | -4.4 | -4.8 | -7.2 | -7.6 | -9.0 | -8.6 | -7.4 | -6.2 | -4.0 | -2.9 | -1.8 | -1.3 | -0.8 |

**Table 1-8: Sit-and-Reach Scoring Table for Girls (Unit: cm)**

| **Level** | **Individual Score** | **Grade 1** | **Grade 2** | **Grade 3** | **Grade 4** | **Grade 5** | **Grade 6** | **Junior 1** | **Junior 2** | **Junior 3** | **Senior 1** | **Senior 2** | **Senior 3** | **Freshman**  **Sophomore** | **Junior**  **Senior** |
| --- | --- | --- | --- | --- | --- | --- | --- | --- | --- | --- | --- | --- | --- | --- | --- |
| **Excellent** | **100** | 18.6 | 18.9 | 19.2 | 19.5 | 19.8 | 19.9 | 21.8 | 22.7 | 23.5 | 24.2 | 24.8 | 25.3 | 25.8 | 26.3 |
|  | **95** | 17.3 | 17.6 | 17.9 | 18.1 | 18.5 | 18.7 | 20.1 | 21.0 | 21.8 | 22.5 | 23.1 | 23.6 | 24.0 | 24.4 |
|  | **90** | 16.0 | 16.3 | 16.6 | 16.9 | 17.2 | 17.5 | 18.4 | 19.3 | 20.1 | 20.8 | 21.4 | 21.9 | 22.2 | 22.4 |
| **Good** | **85** | 14.7 | 14.8 | 14.9 | 15.0 | 15.1 | 15.2 | 16.7 | 17.6 | 18.4 | 19.1 | 19.7 | 20.2 | 20.6 | 21.0 |
|  | **80** | 13.4 | 13.3 | 13.2 | 13.1 | 13.0 | 12.9 | 15.0 | 15.9 | 16.7 | 17.4 | 18.0 | 18.5 | 19.0 | 19.5 |
| **Pass** | **78** | 12.3 | 12.2 | 12.1 | 12.0 | 11.9 | 11.8 | 13.7 | 14.6 | 15.4 | 16.1 | 16.7 | 17.2 | 17.7 | 18.2 |
|  | **76** | 11.2 | 11.1 | 11.0 | 10.9 | 10.8 | 10.7 | 12.4 | 13.3 | 14.1 | 14.8 | 15.4 | 15.9 | 16.4 | 16.9 |
|  | **74** | 10.1 | 10.0 | 9.9 | 9.8 | 9.7 | 9.6 | 11.1 | 12.0 | 12.8 | 13.5 | 14.1 | 14.6 | 15.1 | 15.6 |
|  | **72** | 9.0 | 8.9 | 8.8 | 8.7 | 8.6 | 8.5 | 9.8 | 10.7 | 11.5 | 12.2 | 12.8 | 13.3 | 13.8 | 14.3 |
|  | **70** | 7.9 | 7.8 | 7.7 | 7.6 | 7.5 | 7.4 | 8.5 | 9.4 | 10.2 | 10.9 | 11.5 | 12.0 | 12.5 | 13.0 |
|  | **68** | 6.8 | 6.7 | 6.6 | 6.5 | 6.4 | 6.3 | 7.2 | 8.1 | 8.9 | 9.6 | 10.2 | 10.7 | 11.2 | 11.7 |
|  | **66** | 5.7 | 5.6 | 5.5 | 5.4 | 5.3 | 5.2 | 5.9 | 6.8 | 7.6 | 8.3 | 8.9 | 9.4 | 9.9 | 10.4 |
|  | **64** | 4.6 | 4.5 | 4.4 | 4.3 | 4.2 | 4.1 | 4.6 | 5.5 | 6.3 | 7.0 | 7.6 | 8.1 | 8.6 | 9.1 |
|  | **62** | 3.5 | 3.4 | 3.3 | 3.2 | 3.1 | 3.0 | 3.3 | 4.2 | 5.0 | 5.7 | 6.3 | 6.8 | 7.3 | 7.8 |
|  | **60** | 2.4 | 2.3 | 2.2 | 2.1 | 2.0 | 1.9 | 2.0 | 2.9 | 3.7 | 4.4 | 5.0 | 5.5 | 6.0 | 6.5 |
| **Fail** | **50** | 1.6 | 1.5 | 1.4 | 1.3 | 1.2 | 1.1 | 1.2 | 2.1 | 2.9 | 3.6 | 4.2 | 4.7 | 5.2 | 5.7 |
|  | **40** | 0.8 | 0.7 | 0.6 | 0.5 | 0.4 | 0.3 | 0.4 | 1.3 | 2.1 | 2.8 | 3.4 | 3.9 | 4.4 | 4.9 |
|  | **30** | 0.0 | -0.1 | -0.2 | -0.3 | -0.4 | -0.5 | -0.4 | 0.5 | 1.3 | 2.0 | 2.6 | 3.1 | 3.6 | 4.1 |
|  | **20** | -0.8 | -0.9 | -1.0 | -1.1 | -1.2 | -1.3 | -1.2 | -0.3 | 0.5 | 1.2 | 1.8 | 2.3 | 2.8 | 3.3 |
|  | **10** | -1.6 | -1.7 | -1.8 | -1.9 | -2.0 | -2.1 | -2.0 | -1.1 | -0.3 | 0.4 | 1.0 | 1.5 | 2.0 | 2.5 |

**Table 1-9: 1-minute Rope Skipping Scoring Table for Boys (Unit: times)**

| **Level** | **Individual Score** | **Grade 1** | **Grade 2** | **Grade 3** | **Grade 4** | **Grade 5** | **Grade 6** |
| --- | --- | --- | --- | --- | --- | --- | --- |
| **Excellent** | **100** | 109 | 117 | 126 | 137 | 148 | 157 |
|  | **95** | 104 | 112 | 121 | 132 | 143 | 152 |
|  | **90** | 99 | 107 | 116 | 127 | 138 | 147 |
| **Good** | **85** | 93 | 101 | 110 | 121 | 132 | 141 |
|  | **80** | 87 | 95 | 104 | 115 | 126 | 135 |
| **Pass** | **78** | 80 | 88 | 97 | 108 | 119 | 128 |
|  | **76** | 73 | 81 | 90 | 101 | 112 | 121 |
|  | **74** | 66 | 74 | 83 | 94 | 105 | 114 |
|  | **72** | 59 | 67 | 76 | 87 | 98 | 107 |
|  | **70** | 52 | 60 | 69 | 80 | 91 | 100 |
|  | **68** | 45 | 53 | 62 | 73 | 84 | 93 |
|  | **66** | 38 | 46 | 55 | 66 | 77 | 86 |
|  | **64** | 31 | 39 | 48 | 59 | 70 | 79 |
|  | **62** | 24 | 32 | 41 | 52 | 63 | 72 |
|  | **60** | 17 | 25 | 34 | 45 | 56 | 65 |
| **Fail** | **50** | 14 | 22 | 31 | 42 | 53 | 62 |
|  | **40** | 11 | 19 | 28 | 39 | 50 | 59 |
|  | **30** | 8 | 16 | 25 | 36 | 47 | 56 |
|  | **20** | 5 | 13 | 22 | 33 | 44 | 53 |
|  | **10** | 2 | 10 | 19 | 30 | 41 | 50 |

**Table 1-10: 1-minute Rope Skipping Scoring Table for Girls (Unit: times)**

| **Level** | **Individual Score** | **Grade 1** | **Grade 2** | **Grade 3** | **Grade 4** | **Grade 5** | **Grade 6** |
| --- | --- | --- | --- | --- | --- | --- | --- |
| **Excellent** | **100** | 117 | 127 | 139 | 149 | 158 | 166 |
|  | **95** | 110 | 120 | 132 | 142 | 151 | 159 |
|  | **90** | 103 | 113 | 125 | 135 | 144 | 152 |
| **Good** | **85** | 95 | 105 | 117 | 127 | 136 | 144 |
|  | **80** | 87 | 97 | 109 | 119 | 128 | 136 |
| **Pass** | **78** | 80 | 90 | 102 | 112 | 121 | 129 |
|  | **76** | 73 | 83 | 95 | 105 | 114 | 122 |
|  | **74** | 66 | 76 | 88 | 98 | 107 | 115 |
|  | **72** | 59 | 69 | 81 | 91 | 100 | 108 |
|  | **70** | 52 | 62 | 74 | 84 | 93 | 101 |
|  | **68** | 45 | 55 | 67 | 77 | 86 | 94 |
|  | **66** | 38 | 48 | 60 | 70 | 79 | 87 |
|  | **64** | 31 | 41 | 53 | 63 | 72 | 80 |
|  | **62** | 24 | 34 | 46 | 56 | 65 | 73 |
|  | **60** | 17 | 27 | 39 | 49 | 58 | 66 |
| **Fail** | **50** | 14 | 24 | 36 | 46 | 55 | 63 |
|  | **40** | 11 | 21 | 33 | 43 | 52 | 60 |
|  | **30** | 8 | 18 | 30 | 40 | 49 | 57 |
|  | **20** | 5 | 15 | 27 | 37 | 46 | 54 |
|  | **10** | 2 | 12 | 24 | 34 | 43 | 51 |

**Table 1-11: Standing Long Jump Scoring Table for Boys (Unit: cm)**

| **Level** | **Individual Score** | **Junior 1** | **Junior 2** | **Junior 3** | **Senior 1** | **Senior 2** | **Senior 3** | **Freshman**  **Sophomore** | **Junior**  **Senior** |
| --- | --- | --- | --- | --- | --- | --- | --- | --- | --- |
| **Excellent** | **100** | 225 | 240 | 250 | 260 | 265 | 270 | 273 | 275 |
|  | **95** | 218 | 233 | 245 | 255 | 260 | 265 | 268 | 270 |
|  | **90** | 211 | 226 | 240 | 250 | 255 | 260 | 263 | 265 |
| **Good** | **85** | 203 | 218 | 233 | 243 | 248 | 253 | 256 | 258 |
|  | **80** | 195 | 210 | 225 | 235 | 240 | 245 | 248 | 250 |
| **Pass** | **78** | 191 | 206 | 221 | 231 | 236 | 241 | 244 | 246 |
|  | **76** | 187 | 202 | 217 | 227 | 232 | 237 | 240 | 242 |
|  | **74** | 183 | 198 | 213 | 223 | 228 | 233 | 236 | 238 |
|  | **72** | 179 | 194 | 209 | 219 | 224 | 229 | 232 | 234 |
|  | **70** | 175 | 190 | 205 | 215 | 220 | 225 | 228 | 230 |
|  | **68** | 171 | 186 | 201 | 211 | 216 | 221 | 224 | 226 |
|  | **66** | 167 | 182 | 197 | 207 | 212 | 217 | 220 | 222 |
|  | **64** | 163 | 178 | 193 | 203 | 208 | 213 | 216 | 218 |
|  | **62** | 159 | 174 | 189 | 199 | 204 | 209 | 212 | 214 |
|  | **60** | 155 | 170 | 185 | 195 | 200 | 205 | 208 | 210 |
| **Fail** | **50** | 150 | 165 | 180 | 190 | 195 | 200 | 203 | 205 |
|  | **40** | 145 | 160 | 175 | 185 | 190 | 195 | 198 | 200 |
|  | **30** | 140 | 155 | 170 | 180 | 185 | 190 | 193 | 195 |
|  | **20** | 135 | 150 | 165 | 175 | 180 | 185 | 188 | 190 |
|  | **10** | 130 | 145 | 160 | 170 | 175 | 180 | 183 | 185 |

**Table 1-12: Standing Long Jump Scoring Table for Girls (Unit: cm)**

| **Level** | **Individual Score** | **Junior 1** | **Junior 2** | **Junior 3** | **Senior 1** | **Senior 2** | **Senior 3** | **Freshman**  **Sophomore** | **Junior**  **Senior** |
| --- | --- | --- | --- | --- | --- | --- | --- | --- | --- |
| **Excellent** | **100** | 196 | 200 | 202 | 204 | 205 | 206 | 207 | 208 |
|  | **95** | 190 | 194 | 196 | 198 | 199 | 200 | 201 | 202 |
|  | **90** | 184 | 188 | 190 | 192 | 193 | 194 | 195 | 196 |
| **Good** | **85** | 177 | 181 | 183 | 185 | 186 | 187 | 188 | 189 |
|  | **80** | 170 | 174 | 176 | 178 | 179 | 180 | 181 | 182 |
| **Pass** | **78** | 167 | 171 | 173 | 175 | 176 | 177 | 178 | 179 |
|  | **76** | 164 | 168 | 170 | 172 | 173 | 174 | 175 | 176 |
|  | **74** | 161 | 165 | 167 | 169 | 170 | 171 | 172 | 173 |
|  | **72** | 158 | 162 | 164 | 166 | 167 | 168 | 169 | 170 |
|  | **70** | 155 | 159 | 161 | 163 | 164 | 165 | 166 | 167 |
|  | **68** | 152 | 156 | 158 | 160 | 161 | 162 | 163 | 164 |
|  | **66** | 149 | 153 | 155 | 157 | 158 | 159 | 160 | 161 |
|  | **64** | 146 | 150 | 152 | 154 | 155 | 156 | 157 | 158 |
|  | **62** | 143 | 147 | 149 | 151 | 152 | 153 | 154 | 155 |
|  | **60** | 140 | 144 | 146 | 148 | 149 | 150 | 151 | 152 |
| **Fail** | **50** | 135 | 139 | 141 | 143 | 144 | 145 | 146 | 147 |
|  | **40** | 130 | 134 | 136 | 138 | 139 | 140 | 141 | 142 |
|  | **30** | 125 | 129 | 131 | 133 | 134 | 135 | 136 | 137 |
|  | **20** | 120 | 124 | 126 | 128 | 129 | 130 | 131 | 132 |
|  | **10** | 115 | 119 | 121 | 123 | 124 | 125 | 126 | 127 |

**Table 1-13: 1-minute Sit-up / Pull-ups Scoring Table for Boys (Unit: times)**

| **Level** | **Individual Score** | **Grade 3** | **Grade 4** | **Grade 5** | **Grade 6** | **Junior 1** | **Junior 2** | **Junior 3** | **Senior 1** | **Senior 2** | **Senior 3** | **Freshman**  **Sophomore** | **Junior**  **Senior** |
| --- | --- | --- | --- | --- | --- | --- | --- | --- | --- | --- | --- | --- | --- |
| **Excellent** | **100** | 48 | 49 | 50 | 51 | 13 | 14 | 15 | 16 | 17 | 18 | 19 | 20 |
|  | **95** | 45 | 46 | 47 | 48 | 12 | 13 | 14 | 15 | 16 | 17 | 18 | 19 |
|  | **90** | 42 | 43 | 44 | 45 | 11 | 12 | 13 | 14 | 15 | 16 | 17 | 18 |
| **Good** | **85** | 39 | 40 | 41 | 42 | 10 | 11 | 12 | 13 | 14 | 15 | 16 | 17 |
|  | **80** | 36 | 37 | 38 | 39 | 9 | 10 | 11 | 12 | 13 | 14 | 15 | 16 |
| **Pass** | **78** | 34 | 35 | 36 | 37 |  |  |  |  |  |  |  |  |
|  | **76** | 32 | 33 | 34 | 35 | 8 | 9 | 10 | 11 | 12 | 13 | 14 | 15 |
|  | **74** | 30 | 31 | 32 | 33 |  |  |  |  |  |  |  |  |
|  | **72** | 28 | 29 | 30 | 31 | 7 | 8 | 9 | 10 | 11 | 12 | 13 | 14 |
|  | **70** | 26 | 27 | 28 | 29 |  |  |  |  |  |  |  |  |
|  | **68** | 24 | 25 | 26 | 27 | 6 | 7 | 8 | 9 | 10 | 11 | 12 | 13 |
|  | **66** | 22 | 23 | 24 | 25 |  |  |  |  |  |  |  |  |
|  | **64** | 20 | 21 | 22 | 23 | 5 | 6 | 7 | 8 | 9 | 10 | 11 | 12 |
|  | **62** | 18 | 19 | 20 | 21 |  |  |  |  |  |  |  |  |
|  | **60** | 16 | 17 | 18 | 19 | 4 | 5 | 6 | 7 | 8 | 9 | 10 | 11 |
| **Fail** | **50** | 14 | 15 | 16 | 17 | 3 | 4 | 5 | 6 | 7 | 8 | 9 | 10 |
|  | **40** | 12 | 13 | 14 | 15 | 2 | 3 | 4 | 5 | 6 | 7 | 8 | 9 |
|  | **30** | 10 | 11 | 12 | 13 | 1 | 2 | 3 | 4 | 5 | 6 | 7 | 8 |
|  | **20** | 8 | 9 | 10 | 11 |  | 1 | 2 | 3 | 4 | 5 | 6 | 7 |
|  | **10** | 6 | 7 | 8 | 9 |  |  | 1 | 2 | 3 | 4 | 5 | 6 |

**Note: For grades 3-6 in elementary school: 1-minute sit-ups; for middle school, high school, and university: pull-ups.**

**Table 1-14: 1-minute Sit-up Scoring Table for Girls (Unit: times)**

| **Level** | **Individual Score** | **Grade 3** | **Grade 4** | **Grade 5** | **Grade 6** | **Junior 1** | **Junior 2** | **Junior 3** | **Senior 1** | **Senior 2** | **Senior 3** | **Freshman**  **Sophomore** | **Junior**  **Senior** |
| --- | --- | --- | --- | --- | --- | --- | --- | --- | --- | --- | --- | --- | --- |
| **Excellent** | **100** | 46 | 47 | 48 | 49 | 50 | 51 | 52 | 53 | 54 | 55 | 56 | 57 |
|  | **95** | 44 | 45 | 46 | 47 | 48 | 49 | 50 | 51 | 52 | 53 | 54 | 55 |
|  | **90** | 42 | 43 | 44 | 45 | 46 | 47 | 48 | 49 | 50 | 51 | 52 | 53 |
| **Good** | **85** | 39 | 40 | 41 | 42 | 43 | 44 | 45 | 46 | 47 | 48 | 49 | 50 |
|  | **80** | 36 | 37 | 38 | 39 | 40 | 41 | 42 | 43 | 44 | 45 | 46 | 47 |
| **Pass** | **78** | 34 | 35 | 36 | 37 | 38 | 39 | 40 | 41 | 42 | 43 | 44 | 45 |
|  | **76** | 32 | 33 | 34 | 35 | 36 | 37 | 38 | 39 | 40 | 41 | 42 | 43 |
|  | **74** | 30 | 31 | 32 | 33 | 34 | 35 | 36 | 37 | 38 | 39 | 40 | 41 |
|  | **72** | 28 | 29 | 30 | 31 | 32 | 33 | 34 | 35 | 36 | 37 | 38 | 39 |
|  | **70** | 26 | 27 | 28 | 29 | 30 | 31 | 32 | 33 | 34 | 35 | 36 | 37 |
|  | **68** | 24 | 25 | 26 | 27 | 28 | 29 | 30 | 31 | 32 | 33 | 34 | 35 |
|  | **66** | 22 | 23 | 24 | 25 | 26 | 27 | 28 | 29 | 30 | 31 | 32 | 33 |
|  | **64** | 20 | 21 | 22 | 23 | 24 | 25 | 26 | 27 | 28 | 29 | 30 | 31 |
|  | **62** | 18 | 19 | 20 | 21 | 22 | 23 | 24 | 25 | 26 | 27 | 28 | 29 |
|  | **60** | 16 | 17 | 18 | 19 | 20 | 21 | 22 | 23 | 24 | 25 | 26 | 27 |
| **Fail** | **50** | 14 | 15 | 16 | 17 | 18 | 19 | 20 | 21 | 22 | 23 | 24 | 25 |
|  | **40** | 12 | 13 | 14 | 15 | 16 | 17 | 18 | 19 | 20 | 21 | 22 | 23 |
|  | **30** | 10 | 11 | 12 | 13 | 14 | 15 | 16 | 17 | 18 | 19 | 20 | 21 |
|  | **20** | 8 | 9 | 10 | 11 | 12 | 13 | 14 | 15 | 16 | 17 | 18 | 19 |
|  | **10** | 6 | 7 | 8 | 9 | 10 | 11 | 12 | 13 | 14 | 15 | 16 | 17 |

**Table 1-15: Endurance Run Scoring Table for Boys (Unit: minutes·seconds)**

| **Level** | **Individual Score** | **Grade 5** | **Grade 6** | **Junior 1** | **Junior 2** | **Junior 3** | **Senior 1** | **Senior 2** | **Senior 3** | **Freshman**  **Sophomore** | **Junior**  **Senior** |
| --- | --- | --- | --- | --- | --- | --- | --- | --- | --- | --- | --- |
| **Excellent** | **100** | 1'36" | 1'30" | 3'55" | 3'50" | 3'40" | 3'30" | 3'25" | 3'20" | 3'17" | 3'15" |
|  | **95** | 1'39" | 1'33" | 4'05" | 3'55" | 3'45" | 3'35" | 3'30" | 3'25" | 3'22" | 3'20" |
|  | **90** | 1'42" | 1'36" | 4'15" | 4'00" | 3'50" | 3'40" | 3'35" | 3'30" | 3'27" | 3'25" |
| **Good** | **85** | 1'45" | 1'39" | 4'22" | 4'07" | 3'57" | 3'47" | 3'42" | 3'37" | 3'34" | 3'32" |
|  | **80** | 1'48" | 1'42" | 4'30" | 4'15" | 4'05" | 3'55" | 3'50" | 3'45" | 3'42" | 3'40" |
| **Pass** | **78** | 1'51" | 1'45" | 4'35" | 4'20" | 4'10" | 4'00" | 3'55" | 3'50" | 3'47" | 3'45" |
|  | **76** | 1'54" | 1'48" | 4'40" | 4'25" | 4'15" | 4'05" | 4'00" | 3'55" | 3'52" | 3'50" |
|  | **74** | 1'57" | 1'51" | 4'45" | 4'30" | 4'20" | 4'10" | 4'05" | 4'00" | 3'57" | 3'55" |
|  | **72** | 2'00" | 1'54" | 4'50" | 4'35" | 4'25" | 4'15" | 4'10" | 4'05" | 4'02" | 4'00" |
|  | **70** | 2'03" | 1'57" | 4'55" | 4'40" | 4'30" | 4'20" | 4'15" | 4'10" | 4'07" | 4'05" |
|  | **68** | 2'06" | 2'00" | 5'00" | 4'45" | 4'35" | 4'25" | 4'20" | 4'15" | 4'12" | 4'10" |
|  | **66** | 2'09" | 2'03" | 5'05" | 4'50" | 4'40" | 4'30" | 4'25" | 4'20" | 4'17" | 4'15" |
|  | **64** | 2'12" | 2'06" | 5'10" | 4'55" | 4'45" | 4'35" | 4'30" | 4'25" | 4'22" | 4'20" |
|  | **62** | 2'15" | 2'09" | 5'15" | 5'00" | 4'50" | 4'40" | 4'35" | 4'30" | 4'27" | 4'25" |
|  | **60** | 2'18" | 2'12" | 5'20" | 5'05" | 4'55" | 4'45" | 4'40" | 4'35" | 4'32" | 4'30" |
| **Fail** | **50** | 2'22" | 2'16" | 5'40" | 5'25" | 5'15" | 5'05" | 5'00" | 4'55" | 4'52" | 4'50" |
|  | **40** | 2'26" | 2'20" | 6'00" | 5'45" | 5'35" | 5'25" | 5'20" | 5'15" | 5'12" | 5'10" |
|  | **30** | 2'30" | 2'24" | 6'20" | 6'05" | 5'55" | 5'45" | 5'40" | 5'35" | 5'32" | 5'30" |
|  | **20** | 2'34" | 2'28" | 6'40" | 6'25" | 6'15" | 6'05" | 6'00" | 5'55" | 5'52" | 5'50" |
|  | **10** | 2'38" | 2'32" | 7'00" | 6'45" | 6'35" | 6'25" | 6'20" | 6'15" | 6'12" | 6'10" |

Note: For grades 5-6 in elementary school: 50-meter × 8 shuttle run; for middle school, high school, and university: 1000-meter run.

**Table 1-16: Endurance Run Scoring Table for Girls (Unit: minutes·seconds)**

| **Level** | **Individual Score** | **Grade 5** | **Grade 6** | **Junior 1** | **Junior 2** | **Junior 3** | **Senior 1** | **Senior 2** | **Senior 3** | **Freshman**  **Sophomore** | **Junior**  **Senior** |
| --- | --- | --- | --- | --- | --- | --- | --- | --- | --- | --- | --- |
| **Excellent** | **100** | 1'41" | 1'37" | 3'35" | 3'30" | 3'25" | 3'24" | 3'22" | 3'20" | 3'18" | 3'16" |
|  | **95** | 1'44" | 1'40" | 3'42" | 3'37" | 3'32" | 3'30" | 3'28" | 3'26" | 3'24" | 3'22" |
|  | **90** | 1'47" | 1'43" | 3'49" | 3'44" | 3'39" | 3'36" | 3'34" | 3'32" | 3'30" | 3'28" |
| **Good** | **85** | 1'50" | 1'46" | 3'57" | 3'52" | 3'47" | 3'43" | 3'41" | 3'39" | 3'37" | 3'35" |
|  | **80** | 1'53" | 1'49" | 4'05" | 4'00" | 3'55" | 3'50" | 3'48" | 3'46" | 3'44" | 3'42" |
| **Pass** | **78** | 1'56" | 1'52" | 4'10" | 4'05" | 4'00" | 3'55" | 3'53" | 3'51" | 3'49" | 3'47" |
|  | **76** | 1'59" | 1'55" | 4'15" | 4'10" | 4'05" | 4'00" | 3'58" | 3'56" | 3'54" | 3'52" |
|  | **74** | 2'02" | 1'58" | 4'20" | 4'15" | 4'10" | 4'05" | 4'03" | 4'01" | 3'59" | 3'57" |
|  | **72** | 2'05" | 2'01" | 4'25" | 4'20" | 4'15" | 4'10" | 4'08" | 4'06" | 4'04" | 4'02" |
|  | **70** | 2'08" | 2'04" | 4'30" | 4'25" | 4'20" | 4'15" | 4'13" | 4'11" | 4'09" | 4'07" |
|  | **68** | 2'11" | 2'07" | 4'35" | 4'30" | 4'25" | 4'20" | 4'18" | 4'16" | 4'14" | 4'12" |
|  | **66** | 2'14" | 2'10" | 4'40" | 4'35" | 4'30" | 4'25" | 4'23" | 4'21" | 4'19" | 4'17" |
|  | **64** | 2'17" | 2'13" | 4'45" | 4'40" | 4'35" | 4'30" | 4'28" | 4'26" | 4'24" | 4'22" |
|  | **62** | 2'20" | 2'16" | 4'50" | 4'45" | 4'40" | 4'35" | 4'33" | 4'31" | 4'29" | 4'27" |
|  | **60** | 2'23" | 2'19" | 4'55" | 4'50" | 4'45" | 4'40" | 4'38" | 4'36" | 4'34" | 4'32" |
| **Fail** | **50** | 2'27" | 2'23" | 5'05" | 5'00" | 4'55" | 4'50" | 4'48" | 4'46" | 4'44" | 4'42" |
|  | **40** | 2'31" | 2'27" | 5'15" | 5'10" | 5'05" | 5'00" | 4'58" | 4'56" | 4'54" | 4'52" |
|  | **30** | 2'35" | 2'31" | 5'25" | 5'20" | 5'15" | 5'10" | 5'08" | 5'06" | 5'04" | 5'02" |
|  | **20** | 2'39" | 2'35" | 5'35" | 5'30" | 5'25" | 5'20" | 5'18" | 5'16" | 5'14" | 5'12" |
|  | **10** | 2'43" | 2'39" | 5'45" | 5'40" | 5'35" | 5'30" | 5'28" | 5'26" | 5'24" | 5'22" |

Note: For grades 5-6 in elementary school: 50-meter × 8 shuttle run; for middle school, high school, and university: 800-meter run.

(II) Bonus Points Scoring Tables

**Table 2-1: 1-minute Rope Skipping Scoring Table for Boys (Unit: times)**

| **Bonus Points** | **Grade 1** | **Grade 2** | **Grade 3** | **Grade 4** | **Grade 5** | **Grade 6** |
| --- | --- | --- | --- | --- | --- | --- |
| **20** | 40 | 40 | 40 | 40 | 40 | 40 |
| **19** | 38 | 38 | 38 | 38 | 38 | 38 |
| **18** | 36 | 36 | 36 | 36 | 36 | 36 |
| **17** | 34 | 34 | 34 | 34 | 34 | 34 |
| **16** | 32 | 32 | 32 | 32 | 32 | 32 |
| **15** | 30 | 30 | 30 | 30 | 30 | 30 |
| **14** | 28 | 28 | 28 | 28 | 28 | 28 |
| **13** | 26 | 26 | 26 | 26 | 26 | 26 |
| **12** | 24 | 24 | 24 | 24 | 24 | 24 |
| **11** | 22 | 22 | 22 | 22 | 22 | 22 |
| **10** | 20 | 20 | 20 | 20 | 20 | 20 |
| **9** | 18 | 18 | 18 | 18 | 18 | 18 |
| **8** | 16 | 16 | 16 | 16 | 16 | 16 |
| **7** | 14 | 14 | 14 | 14 | 14 | 14 |
| **6** | 12 | 12 | 12 | 12 | 12 | 12 |
| **5** | 10 | 10 | 10 | 10 | 10 | 10 |
| **4** | 8 | 8 | 8 | 8 | 8 | 8 |
| **3** | 6 | 6 | 6 | 6 | 6 | 6 |
| **2** | 4 | 4 | 4 | 4 | 4 | 4 |
| **1** | 2 | 2 | 2 | 2 | 2 | 2 |

Note: 1-minute rope skipping is a high-priority indicator. Points are added for scores exceeding 100 points, based on the number of times surpassed.

**Table 2-2: 1-minute Rope Skipping Scoring Table for Girls (Unit: times)**

| **Bonus Points** | **Grade 1** | **Grade 2** | **Grade 3** | **Grade 4** | **Grade 5** | **Grade 6** |
| --- | --- | --- | --- | --- | --- | --- |
| **20** | 40 | 40 | 40 | 40 | 40 | 40 |
| **19** | 38 | 38 | 38 | 38 | 38 | 38 |
| **18** | 36 | 36 | 36 | 36 | 36 | 36 |
| **17** | 34 | 34 | 34 | 34 | 34 | 34 |
| **16** | 32 | 32 | 32 | 32 | 32 | 32 |
| **15** | 30 | 30 | 30 | 30 | 30 | 30 |
| **14** | 28 | 28 | 28 | 28 | 28 | 28 |
| **13** | 26 | 26 | 26 | 26 | 26 | 26 |
| **12** | 24 | 24 | 24 | 24 | 24 | 24 |
| **11** | 22 | 22 | 22 | 22 | 22 | 22 |
| **10** | 20 | 20 | 20 | 20 | 20 | 20 |
| **9** | 18 | 18 | 18 | 18 | 18 | 18 |
| **8** | 16 | 16 | 16 | 16 | 16 | 16 |
| **7** | 14 | 14 | 14 | 14 | 14 | 14 |
| **6** | 12 | 12 | 12 | 12 | 12 | 12 |
| **5** | 10 | 10 | 10 | 10 | 10 | 10 |
| **4** | 8 | 8 | 8 | 8 | 8 | 8 |
| **3** | 6 | 6 | 6 | 6 | 6 | 6 |
| **2** | 4 | 4 | 4 | 4 | 4 | 4 |
| **1** | 2 | 2 | 2 | 2 | 2 | 2 |

Note: 1-minute rope skipping is a high-priority indicator. Points are added for scores exceeding 100 points, based on the number of times surpassed.

**Table 2-3: Pull-ups Scoring Table for Boys (Unit: times)**

| **Bonus Points** | **Junior 1** | **Junior 2** | **Junior 3** | **Senior 1** | **Senior 2** | **Senior 3** | **Freshman**  **Sophomore** | **Junior**  **Senior** |
| --- | --- | --- | --- | --- | --- | --- | --- | --- |
| **10** | 10 | 10 | 10 | 10 | 10 | 10 | 10 | 10 |
| **9** | 9 | 9 | 9 | 9 | 9 | 9 | 9 | 9 |
| **8** | 8 | 8 | 8 | 8 | 8 | 8 | 8 | 8 |
| **7** | 7 | 7 | 7 | 7 | 7 | 7 | 7 | 7 |
| **6** | 6 | 6 | 6 | 6 | 6 | 6 | 6 | 6 |
| **5** | 5 | 5 | 5 | 5 | 5 | 5 | 5 | 5 |
| **4** | 4 | 4 | 4 | 4 | 4 | 4 | 4 | 4 |
| **3** | 3 | 3 | 3 | 3 | 3 | 3 | 3 | 3 |
| **2** | 2 | 2 | 2 | 2 | 2 | 2 | 2 | 2 |
| **1** | 1 | 1 | 1 | 1 | 1 | 1 | 1 | 1 |

**Table 2-4: 1-minute Sit-up Scoring Table for Girls (Unit: times)**

| **Bonus Points** | **Junior 1** | **Junior 2** | **Junior 3** | **Senior 1** | **Senior 2** | **Senior 3** | **Freshman**  **Sophomore** | **Junior**  **Senior** |
| --- | --- | --- | --- | --- | --- | --- | --- | --- |
| **10** | 13 | 13 | 13 | 13 | 13 | 13 | 13 | 13 |
| **9** | 12 | 12 | 12 | 12 | 12 | 12 | 12 | 12 |
| **8** | 11 | 11 | 11 | 11 | 11 | 11 | 11 | 11 |
| **7** | 10 | 10 | 10 | 10 | 10 | 10 | 10 | 10 |
| **6** | 9 | 9 | 9 | 9 | 9 | 9 | 9 | 9 |
| **5** | 8 | 8 | 8 | 8 | 8 | 8 | 8 | 8 |
| **4** | 7 | 7 | 7 | 7 | 7 | 7 | 7 | 7 |
| **3** | 6 | 6 | 6 | 6 | 6 | 6 | 6 | 6 |
| **2** | 4 | 4 | 4 | 4 | 4 | 4 | 4 | 4 |
| **1** | 2 | 2 | 2 | 2 | 2 | 2 | 2 | 2 |

Note: Both pull-ups and 1-minute sit-ups are high-priority indicators. Points are added for scores exceeding 100 points, based on the number of times surpassed.

**Table 2-5: 1000-meter Run Scoring Table for Boys (Unit: minutes·seconds)**

| **Bonus Points** | **Junior 1** | **Junior 2** | **Junior 3** | **Senior 1** | **Senior 2** | **Senior 3** | **Freshman**  **Sophomore** | **Junior**  **Senior** |
| --- | --- | --- | --- | --- | --- | --- | --- | --- |
| **10** | -35" | -35" | -35" | -35" | -35" | -35" | -35" | -35" |
| **9** | -32" | -32" | -32" | -32" | -32" | -32" | -32" | -32" |
| **8** | -29" | -29" | -29" | -29" | -29" | -29" | -29" | -29" |
| **7** | -26" | -26" | -26" | -26" | -26" | -26" | -26" | -26" |
| **6** | -23" | -23" | -23" | -23" | -23" | -23" | -23" | -23" |
| **5** | -20" | -20" | -20" | -20" | -20" | -20" | -20" | -20" |
| **4** | -16" | -16" | -16" | -16" | -16" | -16" | -16" | -16" |
| **3** | -12" | -12" | -12" | -12" | -12" | -12" | -12" | -12" |
| **2** | -8" | -8" | -8" | -8" | -8" | -8" | -8" | -8" |
| **1** | -4" | -4" | -4" | -4" | -4" | -4" | -4" | -4" |

**Table 2-6: 800-meter Run Scoring Table for Girls (Unit: minutes·seconds)**

| **Bonus Points** | **Junior 1** | **Junior 2** | **Junior 3** | **Senior 1** | **Senior 2** | **Senior 3** | **Freshman**  **Sophomore** | **Junior**  **Senior** |
| --- | --- | --- | --- | --- | --- | --- | --- | --- |
| **10** | -50" | -50" | -50" | -50" | -50" | -50" | -50" | -50" |
| **9** | -45" | -45" | -45" | -45" | -45" | -45" | -45" | -45" |
| **8** | -40" | -40" | -40" | -40" | -40" | -40" | -40" | -40" |
| **7** | -35" | -35" | -35" | -35" | -35" | -35" | -35" | -35" |
| **6** | -30" | -30" | -30" | -30" | -30" | -30" | -30" | -30" |
| **5** | -25" | -25" | -25" | -25" | -25" | -25" | -25" | -25" |
| **4** | -20" | -20" | -20" | -20" | -20" | -20" | -20" | -20" |
| **3** | -15" | -15" | -15" | -15" | -15" | -15" | -15" | -15" |
| **2** | -10" | -10" | -10" | -10" | -10" | -10" | -10" | -10" |
| **1** | -5" | -5" | -5" | -5" | -5" | -5" | -5" | -5" |

Note: Both 1000-meter and 800-meter runs are low-priority indicators. Points are added for scores below 100 points, based on the number of seconds reduced
